# Reassortant H9N2 Influenza Viruses Containing H5N1-Like PB1 Genes Isolated from Black-Billed Magpies in Southern China

**DOI:** 10.1371/journal.pone.0025808

**Published:** 2011-09-29

**Authors:** Guoying Dong, Cong Xu, Chengmin Wang, Bin Wu, Jing Luo, Hong Zhang, Dale Louis Nolte, Thomas Jude Deliberto, Mingxing Duan, Guangju Ji, Hongxuan He

**Affiliations:** 1 Key Laboratory of Animal Ecology and Conservation Biology, National Research Center For Wildlife Born Diseases, Institute of Zoology, Chinese Academy of Sciences, Beijing, China; 2 National Laboratory of Biomacromolecules, Institute of Biophysics of Chinese Academy of Sciences, Beijing, China; 3 College of Food Science, Southwest University, Chongqing, China; 4 National Wildlife Disease Program, USDA/APHIS/Wildlife Services, United States Department of Agriculture, Fort Collins, Colorado, United States of America; 5 State Key Laboratory of Biomembrane and Membrane Biotechnology, School of Life Sciences, Tsinghua University, Beijing, China; Duke-NUS Graduate Medical School, Singapore

## Abstract

H9N2 influenza A viruses have become endemic in different types of terrestrial poultry and wild birds in Asia, and are occasionally transmitted to humans and pigs. To evaluate the role of black-billed magpies (*Pica pica*) in the evolution of influenza A virus, we conducted two epidemic surveys on avian influenza viruses in wild black-billed magpies in Guangxi, China in 2005 and characterized three isolated black-billed magpie H9N2 viruses (BbM viruses). Phylogenetic analysis indicated that three BbM viruses were almost identical with 99.7 to 100% nucleotide homology in their whole genomes, and were reassortants containing BJ94-like (Ck/BJ/1/94) HA, NA, M, and NS genes, SH/F/98-like (Ck/SH/F/98) PB2, PA, and NP genes, and H5N1-like (Ck/YN/1252/03, clade 1) PB1 genes. Genetic analysis showed that BbM viruses were most likely the result of multiple reassortments between co-circulating H9N2-like and H5N1-like viruses, and were genetically different from other H9N2 viruses because of the existence of H5N1-like PB1 genes. Genotypical analysis revealed that BbM viruses evolved from diverse sources and belonged to a novel genotype (B46) discovered in our recent study. Molecular analysis suggested that BbM viruses were likely low pathogenic reassortants. However, results of our pathogenicity study demonstrated that BbM viruses replicated efficiently in chickens and a mammalian mouse model but were not lethal for infected chickens and mice. Antigenic analysis showed that BbM viruses were antigenic heterologous with the H9N2 vaccine strain. Our study is probably the first report to document and characterize H9N2 influenza viruses isolated from black-billed magpies in southern China. Our results suggest that black-billed magpies were susceptible to H9N2 influenza viruses, which raise concerns over possible transmissions of reassortant H9N2 viruses among poultry and wild birds.

## Introduction

H9N2 subtype influenza viruses, derived from Eurasian and North American major gene pools [1], [2], have widely circulated in poultry and wild birds since its first detection from turkeys in the United States in 1966 [3]. Panorama phylogenetic analysis from our recent study revealed that H9N2 viral genomes include at least 74 lineages (4). Except for CA189-like (Ty/CA/189/66) and WI/1/66-like (Ty/WI/1/66) lineages from North America, several major lineages of H9N2 isolates from Eurasia [i.e., BJ94-like (Ck/BJ/1/94), G1-like (Qa/HK/G1/97), DE113-like (Dk/DE/113/95), KR323-like (Ck/KR/96323/96), G9-like (Ck/HK/G9/97), SH/F/98-like (Ck/SH/F/98), Y439-like (Dk/HK/Y439/97), and H5N1-like (Ck/YN/1252/03)] have become established in domestic birds [2], [5], [6]. BJ94-like viruses are prevalent primarily in chicken [5], and transmit back to aquatic poultry (mainly domestic duck) [6], or co-circulate with G1-like viruses in quail to generate multiple reassortant variants or genotypes [7]. Similar viruses were also isolated from pigs in Hong Kong and Shandong of China [8], [9]. G1-like viruses are predominant in quail [5], which may have played an important role as a potential intermediate host in the introduction of influenza viruses from aquatic birds to terrestrial poultry [10]. These viruses frequently reassort with either BJ94-like or H5N1-like viruses, forming a complex two-way transmission system between quail and other types of poultry [7]. In 1997, Dk/HK/Y439/97 (Y439-like) was isolated in domestic ducks and phylogenetic analysis indicated that this virus grouped with similar viruses found in chickens [11]. KR323-like viruses, possibly derived from migratory ducks, formed a unique cluster and were responsible for the outbreaks in Korean chicken flocks in 1996 and 1999 [11], [12].

Furthermore, G1-like H9N2 viruses (e.g. HK/1073/99 or HK/1074/99) have been detected in humans since 1999 which caused mild disease symptoms [13], [14]; and six internal genes of these viruses are closely related to the lethal 1997 Hong Kong H5N1 viruses [5]. It was suggested that the H5N1 avian influenza viruses that directly infected humans in Hong Kong in 1997, causing six fatal cases out of 18 infected patients [13], [15], were probably reassortants that acquired their six internal genes from Qa/HK/G1/97 or one of its precursors [5].

It is noteworthy that H9N2 influenza viruses have been isolated from a variety of birds, including pheasant, chukkar, partridge, guinea fowl, and pigeon in the past two decades [16]. In North America, the presence of H9N2 subtype viruses has been identified in wild ducks and shorebirds since 1966 [3], [17]. H9N2 viruses have also been isolated frequently from waterfowl and poultry in Europe, but have rarely been detected in wild birds [18]. In Asia, H9N2 subtype viruses have become endemic in different types of terrestrial poultry and wild birds since the late 1990s [2]. Southern China including Guangxi and Hong Kong in particular has the complex influenza virus ecosystem and may be an influenza epicenter, where the H9N2 viruses causing human infection were most likely directly from live bird market [6], [7], [19] and different subtypes of influenza viruses are co-circulating widely in domestic poultry and wild birds. Moreover, some H9N2 viruses from southern China possessed the propensity to transmit directly from poultry to humans and would be potential hazards against humans [20].

Outbreaks of H9N2 influenza viruses in domestic poultry have resulted in huge economic losses. And human infections by H9N2 viruses reported in 1999 [5] and 2003 [21] have raised concerns on the potential for H9N2 viruses to become pandemic strains. The findings that different subtype avian influenza viruses such as H7N7 (*e.g.* A/magpie/Korea/YJD174/2007) and H5N1 (*e.g*. A/common magpie/Hong Kong/645/2006, A/pied magpie/Liaoning/7/2006, and A/blue magpie/Hong Kong/1993/2007) can be isolated from magpies indicate that magpies may play an important role in the evolution and ecology of influenza A virus. Black-billed magpies (BbM) are very common birds in southern China and have lived in close proximity to human settlements and poultry farms for centuries. In order to evaluate the role of black-billed magpies in the evolution of influenza A viruses, we characterized three H9N2 influenza viruses isolated from wild black-billed magpies during two epidemic surveys of influenza viruses in Guangxi, China in 2005. Eight complete gene segments of isolated BbM viruses were amplified, sequenced and analyzed phylogenetically; and their molecular properties and evolutionary characteristics were determined. In addition, their antigenicity, replication and pathogenicity in chickens and mice were also investigated.

## Results

### Virus isolation

A total of 32 paired tracheal and cloacal swabs were collected from black-billed magpies living in the wild bird reservoir in Guangxi of China in 2005. Collected samples were placed into viral transport media and maintained at −70°C until they were inoculated into 10-day-old specific-pathogen-free (SPF) embryonated chicken eggs. Three H9N2 subtype in fluenza viruses were isolated from three separate birds among 32 black-billed magpies (isolation rate 9.4%), and were named A/Black-billed Magpie/Guangxi/29/2005 (BbM/GX/29/05) to A/Black-billed Magpie/Guangxi/31/2005 (BbM/GX/31/05). Of these BbM viruses, BbM/GX/29/05 was isolated from the cloacal swab, while BbM/GX/30/05 and BbM/GX/31/05 were isolated from the tracheal samples.

### Phylogenetic analysis of the surface genes

**Figure 1:**
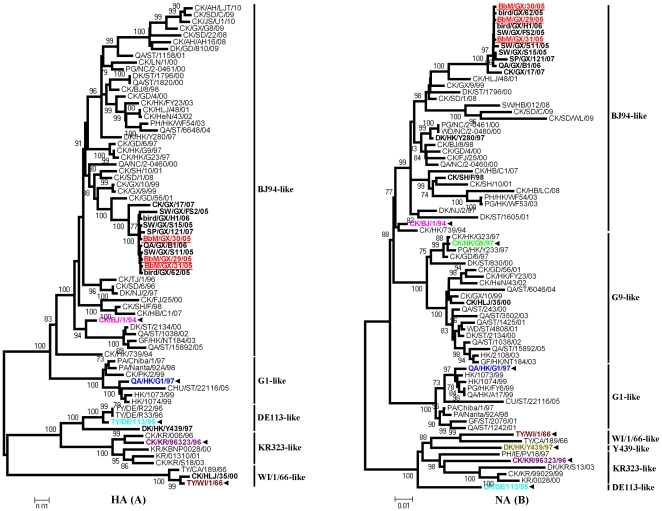
Phylogenetic relationships for HA (A) and NA (B) genes of the BbM viruses and analyzed H9N2 reference viruses. Neighbor-joining phylogenetic trees were generated using the MEGA program. Nucleotides 82 to 1396 for HA and 41 to 1363 for NA genes were analyzed. BbM viruses were underlined in red, while representative viruses from each lineage were highlighted in other colors and marked using bold black arrow. Abbreviations: BbM, black-billed magpie; CHU and CU, chukkar; CK, chicken; DK, duck; GF, guinea fowl; GS, goose; PA, Parakeet; PG, pigeon; PH, pheasant; QA, quail; SP, sparrow; SW, swine; TY, turkey; WD, wild duck; AH, Anhui; BJ, Beijing; CA, California; DE, Germany; FJ, Fujian; GD, Guangdong; GX, Guangxi; HB, Hubei; HK, Hong Kong; HLJ, Heilongjiang; HeN, Henan; IE, Ireland; JS, Jiangsu; KR, Korea; LN, Liaoning; NC, Nanchang; NJ, Nanjing; PK, Pakistan; SD, Shandong; SH, Shanghai; ST, Shantou; TB, Tibet; TJ, Tianjin; WI, Wisconsin; and YU, Yunnan.

Phylogenetic analysis of HA genes showed that H9N2 viruses were divided into two distinctive clusters with similar branching patterns as previously reported [6]. The North American cluster was represented by Ty/WI/1/66 (WI/1/66-like lineage), while the Eurasian cluster comprised four lineages represented by Ck/BJ/1/94 (BJ94-like), Qa/HK/G1/97 (G1-like), Ty/DE/113/95 (DE113-like) and Ck/KR/96323/96 (KR323-like) respectively (Figure 1A). The HA genes of BbM viruses (1742 nucleotides) shared 93.8% to 94.1% nucleotide homology with Ck/BJ/1/94, and formed a single branch with 2005-2007 Guangxi isolates (nucleotide similarity 97.7% to 100%). The BbM viruses clustered within the BJ94-like lineage in the HA phylogenetic tree together with most H9N2 isolates from chickens, ducks, pigeons, quail and some H9N2 pig and wild bird isolates from China. These isolates showed gradual evolution over the last several years and were closely related to Ck/HK/739/94 sharing 95.6 to 99.2% nucleotide homology with Ck/BJ/1/94 in its whole genome. Although they were closely related to the Eurasian cluster, the BbM viruses were distinguishable from G1-like, KR323-like, and DE113-like viruses. In contrast, the HA genes of BbM viruses were significantly different from that of Ck/HLJ/35/00 (about 78.5% nucleotide similarity), which originated from Ty/WI/1/66 and belonged to the North American lineage [22].

Phylogenetic analysis of NA genes revealed a similar evolutionary pattern to that of HA genes (Figure 1B). NA genes of BbM viruses belonged to the BJ94-like lineage containing Dk/Hk/Y280/97, Ck/SH/F/98, and 2005-2007 Guangxi isolates, and had a 9-nt deletion at positions 206 to 214 in the stalk similar to most BJ94-like viruses. However, BbM viruses were quite different from other H9N2 lineage viruses in NA genes. For example, G9-like viruses had full-length NA genes, G1-like viruses were mainly from humans and quail and clustered into an independent clade. Alternatively, isolates from Korea shared relatively high nucleotide similarity with Ty/WI/1/66 (88.8% to 90.2%) or Dk/HK/Y439/97 (89.2% to 90.9%) and grouped in the branch of the North American lineage. Our phylogenetic analysis of surface genes suggested that BbM viruses and BJ94-like viruses shared common ancestors.

### Phylogenetic analysis of the internal genes

**Figure 2:**
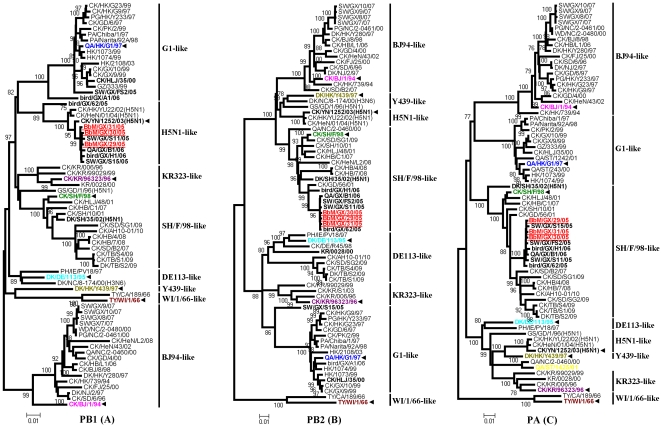
Phylogenetic relationships of PB1 (A), PB2 (B), and PA (C) genes of the BbM viruses and analyzed H9N2 reference viruses. Neighbor-joining phylogenetic trees were generated using the MEGA program. Analysis was based on the following nucleotides: PB1, 229 to 2233; PB2, 52 to 2255; and PA, 58 to 2094. Viruses were labeled using the same way as Figure 1. Virus abbreviations are listed in the legend of Figure 1.

The phylogenetic tree of PB1 genes was separated into eight distinct evolutionary lineages, including G1-like, H5N1-like, KR323-like, SH/F/98-like, DE113-like, Y439-like, WI/1/66-like, and BJ94-like (Figure 2A). Most of H9N2 viruses clustered into the BJ94-like, SH/F/98-like, KR323-like, G1-like and DE113-like lineages. In contrast, the PB1 genes of BbM viruses fell into the H5N1-like lineage, exhibiting the highest nucleotide similarity of 99.3% with that of clade 1 H5N1 isolate Ck/YN/1252/03 [23], and forming a separate fork with most 2005-2006 Guangxi reference strains. These results indicated that the PB1 genes of BbM viruses were probably derived H5N1 viruses instead of other H9N2 isolates.

In the phylogenetic tree of PB2 genes, there were eight different lineages: BJ94-like, Y439-like, H5N1-like, SH/F/98-like, DE113-like, KR323-like, G1-like, and WI/1/66-like (Figure 2B). The BbM viruses clustered into the SH/F/98-like lineage together with 2005–2006 Guangxi isolates except for Sw/GX/S15/05 and bird/GX/A1/06, and were closely related to 2002–2004 H5N1-like viruses (92.7% to 97.1% homology). The clade 0 H5N1 isolate Dk/SH/35/02 [23] was found to be most closely related to BbM viruses with the highest nucleotide similarity of 97.1%. Moreover, SH/F/98-like viruses including BbM viruses formed an independent branch that related to H3N6 representative strain belonging to the Y439-like lineage.

Phylogenetic analysis of PA genes showed that at least eight different lineages were recognized (BJ94-like, G1-like, SH/F/98-like, DE113-like, H5N1-like, Y439-like, KR323-like, and WI/1/66-like) (Figure 2C). The BbM viruses clustered into the SH/F/98-like lineage similar to contemporary Guangxi strains, and were also closely related to the H5N1 isolate Dk/SH/35/02 (95% homology). However, they shared a lower homology (<90.2%) with other H5N1-like viruses.

**Figure 3:**
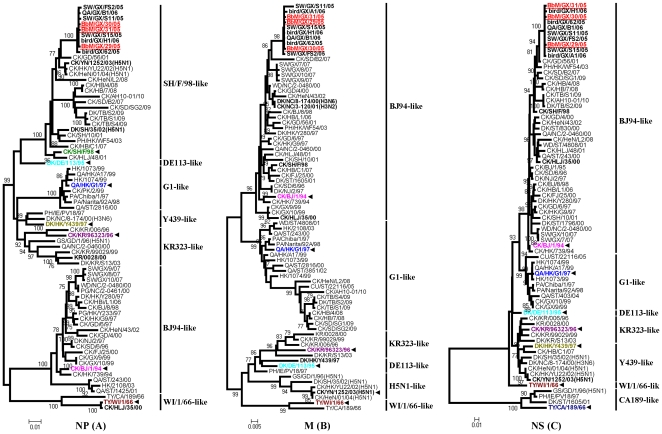
Phylogenetic relationships of NP (A), M (B), and NS (C) genes of the BbM viruses and analyzed H9N2 reference viruses. With MEGA, neighbor-joining phylogenetic trees were constructed based on nucleotides 65 to 1398 for NP, 78 to 784 for M, and 54 to 680 for NS genes. Virus abbreviations are listed in the legend of Figure 1. Viruses were highlighted using the same colors and method as Figure 1.

The phylogenetic tree of NP genes had seven distinct lineages comprised of SH/F/98-like, DE113-like, G1-like, Y439-like, KR323-like, BJ94-like and WI/1/66-like (Figure 3A). The NP genes of BbM viruses shared very high homology (96.9% to 97.8%) with those of 2002–2004 H5N1-like viruses (e.g. Dk/SH/35/02), forming a separate cluster rooted to Ck/SH/F/98 which had an NP gene segment detected initially in an H5N1 virus in 2001 [24]. Particularly, the NP gene of Ck/HLJ/35/00 was apparently derived from Ty/WI/1/66 (99.9% homology) and belonged to the North American lineage. Phylogenetic analyses of PB2, PA and NP genes indicated that the BbM viruses derived from SH/F/98-like viruses and had also a highly close evolutionary relationship to H5N1-like viruses.

In the phylogenetic tree of M genes, the BbM viruses grouped with the BJ94-like lineage containing Ck/SH/F/98 and Ck/HLJ/35/00 (Figure 3B), and were highly related to H3 subtype representative isolates Ck/NC/3-120/01 and Dk/NC/8-174/00 (both sharing 98.1% nucleotide homology). The remaining H9N2 reference viruses used in this study grouped with G1-like, KR323-like, DE113-like, and WI/1/66-like viruses.

For the NS genes, BbM viruses were almost identical in comparison with 2005-2006 Guangxi isolates (99.7% to 100% homology). All these isolates belonged to the BJ94-like lineage and shared a common ancestor with Ck/SH/F/98 (Figure 3C). These results further suggested that the BbM viruses were highly related to BJ94-like and SH/F/98-like viruses.

### Genetic analysis

**Figure 4:**
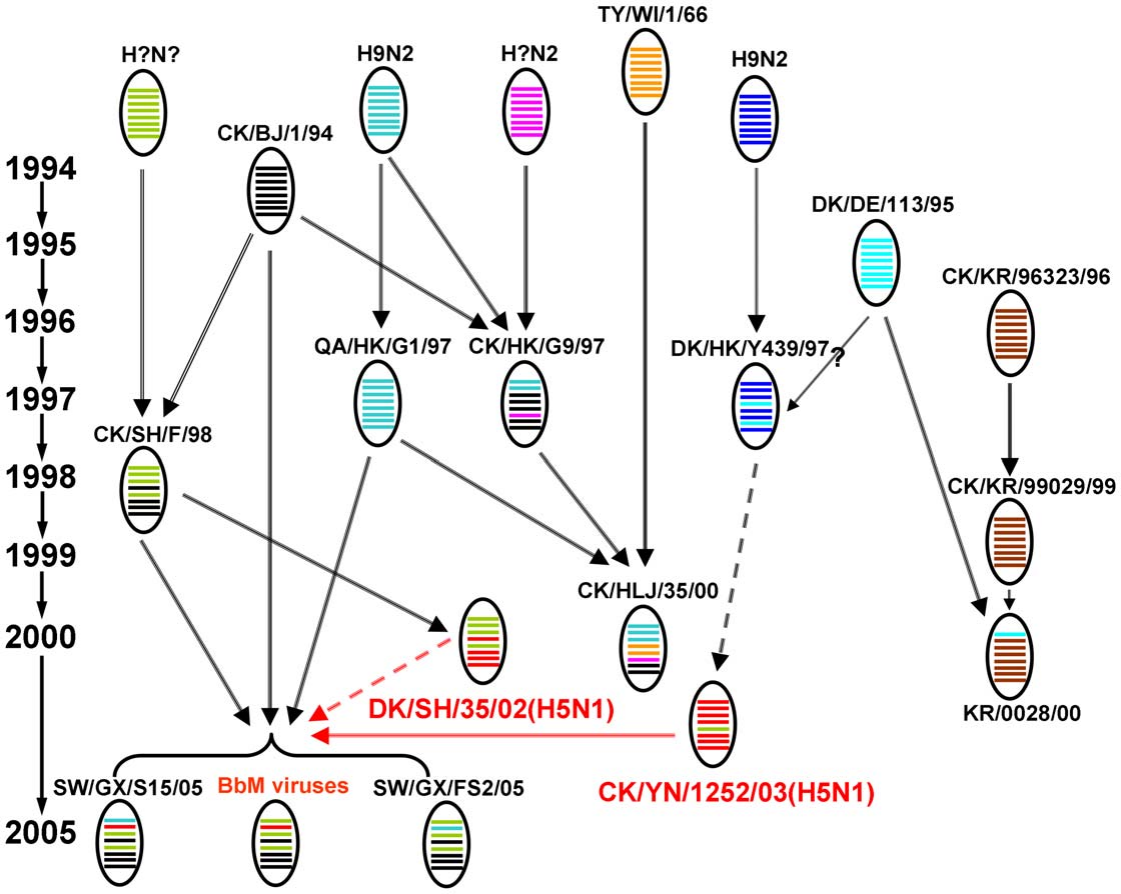
The genetic analysis of BbM viruses and H9N2 reference strains from various lineages. Schematic diagrams represent the origins of genes of BbM viruses and H9N2 reference strains. Eight gene segments in each of the schematic virus particles are arranged from top to bottom to represent PB2, PB1, PA, HA, NP, NA, M, and NS genes, and are indicated in same color with representative viruses for each lineage. Virus names and abbreviations are listed in the legend of Figure 1.

To further determine the genetic characterization and probable origin, the cladogram of BbM viruses and H9N2 representative strains from various lineages was genetically analyzed (Figure 4). The results revealed that BbM viruses were reassortants with the PB1 gene most closely related to the corresponding gene from Ck/YN/1252/03 (H5N1) (the highest degree of similarity 99.3%), similar to Sw/GX/S15/05. The PB2, PA and NP genes of BbM viruses most likely originated from homologous genes of Ck/SH/F/98 (H9N2), and were found to be most closely related to those of the H5N1 Dk/SH/35/02 isolate. The remaining four genes (HA, NA, M and NS) of BbM viruses were all derived from those of the classical H9N2 virus Ck/BJ/1/94. These findings suggested that the BbM viruses most likely arose from reassortments of H9N2 strains and H5N1 viruses, and were genetically different from other H9N2 viruses because of the existence of H5N1-like PB1 genes. Similarly, there were reassortant viruses carrying evolutionarily distant gene segments among other lineages of H9N2 isolates (Figure 4). For example, Ck/HLJ/35/00 reassorted HA and NP genes from Ty/WI/1/66 and the PA gene from Qa/HK/G1/97, on the basis of PB2, PB1, NA, M and NS genes of Ck/Hk/G9/97. The above analysis indicated that H9N2 influenza viruses have undergone extensive and frequent genetic reassortment mainly in China since 1997, and were closely related to H5N1-like viruses in the ribonucleoprotein complex genes.

### Genotypical analysis

Panorama genotypical analysis from our recent study revealed that H9N2 influenza viruses include at least 98 genotypes, which were further divided according to their HA lineages into seven series, designated as A–G representing HK/G1/97, BJ/1/94, KR/96323/96, DE/113/95, HK/289/78, HK/AF157/92, and WI/1/66 lineage viruses, respectively [4]. To further confirm the genetic and evolutionary characteristics of BbM viruses, the genotypical analysis was performed according to available gene constellations of different H9N2 genotypes [4], together with some H9N2 reference strains (Table 1). Results showed that BbM viruses were classified into the genotype B46, the first genotype in which H5N1-like PB1 genes were present [4]. This novel genotype was descended from the SH/F/98-like lineage and likely the result of multiple reassortments between co-circulating H9N2 and H5N1 viruses. Consequently the BbM viruses evolved from diverse sources and belonged to a novel genotype (B46) discovered in our recent study.

**Table 1:** Comparison of amino acid sequences of HAs and NAs of the BbM viruses and H9N2 representative viruses from different lineages.

virus^a^	Genotype	Connecting peptide	RBS^b^ (H3 number)				NA amino acid deletion
			183	190	226	228	
Ck/GD/56/01	B28	R-L-S-R	N	A	L	G	
Ck/HLJ/48/01	B3	R-S-S-R	N	A	L	G	63–65
Ck/SH/F/98	B3	R-S-S-R	N	A	Q	G	63–65
Ck/HK/739/94	B0	R-S-S-R	H	A	Q	G	
Ck/BJ/1/94	B0	R-S-S-R	N	V	Q	G	
Ck/FJ/25/00	B0	R-S-S-R	N	V	L	G	58–70
Pg/NC/2-0461/00	B0	R-S-S-R	N	V	L	G	63–65
Ck/GD/4/00	B0	R-S-S-R	N	T	L	G	63–65
Sw/GX/FS2/05	B50	R-A-S-R	N	A	M	G	63–65
Bird/GX/H1/06	B46	R-A-S-R	N	T	L	G	63–65
Bird/GX/62/05	B46	R-A-S-R	N	T	Q	G	63–65
Sw/GX/S11/05	B46	R-A-S-R	N	T	Q	G	63–65
Qa/GX/B1/06	B46	R-A-S-R	N	T	Q	G	63–65
BbM/GX/29/05	B46	R-A-S-R	N	T	Q	G	63–65
BbM/GX/30/05	B46	R-A-S-R	N	V	Q	G	63–65
BbM/GX/31/05	B46	R-A-S-R	N	T	Q	G	63–65
Sw/GX/S15/05	B51	R-A-S-R	N	V	Q	G	63–65
Ck/GX/9/99	B8	R-S-S-R	N	T	Q	G	63–65
Qa/HK/G1/97	A0	R-S-S-R	H	E	L	G	38–39
Hk/1073/99	A0	R-S-S-R	H	E	L	G	38–39
KR/0028/00	C1	A-S-G-R	H	E	Q	G	64–79
Ty/WI/1/66	G0	V-S-S-R	H	E	Q	G	
Ck/HLJ/35/00	G2	V-S-S-R	H	E	Q	G	
Dk/DE/113/95	D1	A-S-A-R	H	E	Q	G	57–81

a Virus abbreviations are in the legend to Figure 1,

b Amino acid at the receptor binding site.

### Molecular characterization

The deduced amino acid sequences of BbM virus proteins were also compared with those of H9N2 reference viruses. Results indicated that the HAs of BbM viruses shared the same R-A-S-R amino acid motif at the connecting peptide between HA1 and HA2 with those of 2005-2006 H9N2 Guangxi isolates from different hosts (Table 1). Alternatively, the HA connecting peptides of most H9N2 viruses have a R-S-S-R motif. It is noteworthy that the BbM viruses had an S to A substitution at position -3 of the HA1 proteins and a few substitutions were also observed at each site of the connecting peptide in other H9N2 viruses (Table 1). All, though, were predicted to be low pathogenic influenza viruses [25]. Moreover, the HAs of BbM viruses had the conserved avian receptor binding site (RBS) sequences N, V, Q and G at positions 183, 190, 226 and 228, respectively (H3 numbering), with a V to T amino acid substitution at position 190 in BbM/GX/29/05 and BbM/GX/31/05 (Table 1) [26]. The presence of the avian-like motif 226Q suggested that the BbM viruses bind to cellular receptors with 2,3-NeuAcGal linkages [20], [27]. In addition, the amino acid sequences analysis of NA proteins demonstrated that the BbM viruses contained a three-amino acid deletion at positions 63 to 65 in the NA stalk region, similar to the genotype B series viruses but different from the genotype A, C, and D series representative viruses, which have amino acid deletions at positions 38 to 39, 64 to 79, and 57 to 81, respectively (Table 1). The potential biological significance of this molecular marker is thought to be related to the efficiency by which virsuses spread in terrestrial domestic poultry [28]. This finding implied that BbM viruses shared a common genetic property with genotype B series viruses such as Ck/SH/F/98 H9N2 isolate.

According to NetNGlyc 1.0 Server predicted results, eight N-linked potential glycosylation sites (PGS) were conserved in HA proteins of BbM viruses (Figure S1 A1), of which six PGS were in HA1 (29, 82, 141, 218, 298 and 305) (Figure S2) and two in HA2 (492 and 551), similar to many H9N2 viruses [22]. However, BbM viruses were different from Ck/HK/739/94 and Dk/NJ/2/97, because both of them lost a PGS at positions 82 and 218 respectively (Figure S1 A1–A3). The BbM viruses also had seven potential glycosylation sites located on NA proteins at positions 44, 69, 86, 146, 200, 234 and 402 respectively (Figure S1 B1), while amino acid deletions at positions 63 to 65 led to a loss of the related PGS at position 61 in comparison with Ck/BJ/1/94 (Figure S1 B2). Notably, BbM viruses had an extra PGS at position 86 compared to Ck/HLJ/35/00 (Figure S1 B3). Furthermore, BbM viruses had D at position 92 of NS1 proteins, which is typically observed in avian influenza viruses [29], and also possessed E and D at position 627 and 701 of PB2 proteins, which were reportedly associated with increased virulence in pigs and mice, respectively [29], [30]. Specially, BbM viruses were consistent with H5N1-like viruses (e.g. Ck/YN/1252/03) in amino acid residues of the PB1 protein except for two mutations of K387R and K757N (Table S2), and did not have the Y436H amino acid mutation associated with the pathogenicity of H5N1 influenza virus in ducks [31]. Amino acid analysis of M2 proteins showed that BbM viruses did not have any single mutations at amino acids 26L, 27V, 30A, 31S, 34G, 37H, or 41W in the transmembrane region, suggesting that the BbM viruses were still sensitive to amantadines [32], [33].

### Pathogenicity studies

To assess the pathogenicity of BbM viruses in chickens and mice, 6-week-old, SPF chickens and 8-week-old, SPF BALB/c mice were inoculated intranasally (i.n.). Virus replication, morbidity, and mortality were assessed (Table 2). Results of chicken experiments indicated that none of BbM viruses induced clinical signs or deaths during the observation period. However, shedding viruses were detected from tracheal and cloacal swabs of inoculated chickens on day 3 post-inoculation (p.i.) with titers ranging from 1.2 to 4.3 log_10_EID_50_/ml. Results of mouse experiments demonstrated that infection by BbM viruses induced 5% to 10% weight loss of the inoculated mice and BbM viruses could be isolated from lung tissues of infected mice on day 3 p.i. with titers of 4.5 to 5.2 log_10_EID_50_/ml. However, no virus was recovered from spleens and brains and no deaths were observed in inoculated mice during the experiment period. These findings showed that BbM viruses replicated efficiently in chickens and mice but were not lethal to them.

**Table 2:** Results of animal infective experiments with isolated BbM H9N2 in fluenza viruses.

Virus^a^	Chicken infection^b^			Mouse infection^c^			
	No. dead/no. inoculated	Virus in cloaca(log_10_EID_50_)	Virus in trachea(log_10_EID_50_)	No. dead/no. inoculated	Virus in lung(log_10_EID_50_)	Virus in spleen(log_10_EID_50_)	Virus in brain(log_10_EID_50_)
BbM/GX/29/05	0/10	1.8±0.35^d^	4.3±0.33	0/8	5.2±0.22	No	No
BbM/GX/30/05	0/10	1.2±0.44	3.4±0.17	0/8	4.5±0.19	No	No
BbM/GX/31/05	0/10	1.3±0.56	4.2±0.46	0/8	4.8±0.35	No	No

a H9N2 influenza viruses isolated from wild black-billed magpies in this study.

b Groups of ten 6-week-old, SPF chickens were i.n. inoculated with 0.1 ml 10^6.0^ EID_50_ of BbM H9N2 viruses and observed for 21 days for signs of disease. Collected cloacal and tracheal swab samples on day 3 p.i. were titrated for virus infectivity in 10-day-old SPF embryonated chicken eggs.

c Eight 8-week-old SPF female BALB/c mice for each group were i.n. inoculated with 0.05 ml 10^6.0^ EID_50_ of BbM H9N2 viruses and observed daily for 14 days. Tissue samples were collected on day 3 p.i. for virus titration in SPF embryonated chicken eggs.

d Virus titers are given in units of log_10_EID_50_ per 1 ml ± standard deviation (SD).

### Antigenic analysis

The antigenic properties of H9N2 in fluenza viruses from black-billed magpies were investigated with post-infection chicken antisera from three different viruses by HI assay (Table 3). Numerical analysis of HI titers was conducted to visualize the antigenic variation. Results showed BbM H9N2 in fluenza viruses were antigenic heterologous with the H9N2 vaccine strain Ck/SD/6/96 and early isolate Ck/HLJ/35/00 because their HI titers were fourfold lower than the homologous titers. In contrast, the antisera to Ck/GX/10/99 reacted equally well with three BbM H9N2 viruses, suggesting that they were antigenically homologous. These findings further revealed the antigenic diversity of H9N2 in fluenza viruses from China.

**Table 3:** Antigenic analysis of BbM H9N2 influenza viruses.

Virus^a^	HI titers with post-infection antisera^b^		
	Ck/HLJ/35/00	Ck/GX/10/99	Ck/SD/6/96
BbM/GX/29/05	20	320	10
BbM/GX/30/05	20	320	10
BbM/GX/31/05	20	320	10
Ck/HLJ/35/00	160	20	<^c^
Ck/GX/10/99	20	640	<
Ck/SD/6/96	10	160	160

a Virus names and abbreviations are in the legend to Figure 1.

b Antisera were 10-fold diluted.

c <, Below the level of detection of 10.

## Discussion

Given the capability for H9N2 influenza viruses to infect wild birds, poultry, and humans in Asia, further evaluation of potential reservoir species such as black-billed magpies in the evolution of influenza viruses is necessary. Characterization of three H9N2 influenza viruses in this study, isolated firstly from wild black-billed magpies (Passeriformes, Corvidae, a terrestrial bird), revealed that these BbM viruses were circulating in Guangxi of China in 2005. They represent novel genotypical reassortants, containing BJ94-like, SH/F/98-like, and H5N1-like gene segments and substituting for previous SH/F/98-like influenza viruses (Figure 4). Analysis of the viruses phylogeny indicated that the BbM viruses might be initially generated in local chickens, prior to being transmitted to black-billed magpies. The genetic characterization of BbM viruses was remarkably different from those of other H9N2 viruses, especially G1-like and BJ94-like lineage viruses which have been established in terrestrial poultry of southern China since the mid-1990s [5], implying that these natural reassortments occurred independently. Notably, viruses with identical genotype as BbM viruses (i.e., Sw/GX/S11/05, Qa/GX/B1/06 and Bird/GX/H1/06) also emerged in other hosts in the same region (Table 1). Consequently, it could be seen that novel reassortant H9N2 viruses were prevalent in diverse hosts in Guangxi of China during 2005–2006, giving rise to great uncertainty for the current ecosystem of in fluenza viruses in southern China.

Results of phylogenetic analysis demonstrated that HA, NA, M and NS genes of BbM viruses were closely related to those of BJ94-like viruses. Three internal genes, PB2, PA and NP, were descended from SH/F/98-like viruses which may have been the donor of H5N1-like inner genes [7], and were found to be most closely related to those of the H5N1 isolate Dk/SH/35/02. However, the PB1 genes of BbM viruses were originated from H5N1-like viruses exactly as Ck/YN/1252/03 (Figures 1, 2, 3, 4). Consequently, these BbM viruses were likely the result of multiple reassortments between co-circulating H9N2-like (Ck/BJ/1/94 and Ck/SH/F/98) and H5N1-like (Ck/YN/1252/03 and Dk/SH/35/02) viruses, and had highly close evolutionary relationships to H5N1-like viruses in ribonucleoprotein complex genes. The similar evolutionary behavior was also found in the H9N2 isolate Sw/GX/S15/05, which contained gene fragments derived from BJ94-like and SH/F/98-like lineage viruses, and introduced the G1-like PB2 gene and H5N1-like PB1 gene (Figure 4). These findings suggested that complicated reassortments were very prevalent and genetic diversities were extraordinarly evident among reported H9N2 in fluenza viruses [34].

Results of genotypical analysis showed that BbM viruses represent genotype B46 viruses, which documented the existence of H5N1-like PB1 genes, resulting in a single cluster in the PB1 phylogenetic tree and a unique genotype. Genotype B46 virus was first recognized in chickens in 2004 and became predominant in poultry, pigs, and birds during 2005-2006 [4]. These novel genotypical H9N2 reassortants are constantly evolving in diverse hosts, raising the risk of H9N2 influenza viruses to be introduced into humans.

Molecular analysis demonstrated that all BbM viruses maintained the similar HA connecting peptide motifs with terrestrial poultry viruses of low pathogenicity in chicken (Table 1), and also exhibited the same NA stalk deletions as current prevailing chicken isolates, suggesting that the low pathogenic BbM viruses might be transmitted to black-billed magpies from chickens after circulation in chickens for a certain period. Glutamine was conserved at position 226 in the receptor-binding site of HA proteins and position 627 in PB2 proteins, while aspartic acid was conserved at position 701 in PB2 proteins. Conservation of three amino acids is important in determining host range of in fluenza A viruses [35], [36], [37]. These findings further revealed that even though many novel reassortants have been generated, relative host restriction is present for H9N2 viruses [16]. In addition, the analytical results of M2 protein indicated that BbM viruses, not bearing any single substitution at reported amino acid residues crucial for drug resistance [32], [33], should be sensitive to amantadines, which have been frequently used for the influenza treatment in humans and also for H9N2 avian influenza treatment in some areas in China [22].

It was reported recently that Qa/HK/G1/97 replicated efficiently in the human alveolar epithelial A549 cells whereas other H9N2 viruses, Dk/HK/Y280/97 and Ck/HK/G9/97, replicated poorly [38]. Results of pathogenicity studies in chickens and mice indicated that BbM viruses were low pathogenic to chickens and exhibited the same replication phenotype in mice. These viruses were able to replicate in mouse lungs efficiently without any prior adaptation, but none of them was lethal, although they could induce 5% to 10% weight loss of inoculated mice. Currently, most of the avian influenza viruses are restricted in the avian species. The transmissible mechanism of avian in fluenza viruses to mammals is not resolved and was proposed to involve multiple viral genes. The mechanism of transmission and adaptation of BbM viruses to mammals need to be further studied.

The aquatic birds including ducks, shorebirds, and gulls have been regarded as the natural reservoir for in fluenza A virus, with sixteen HA and nine NA subtypes reported to date [1]. However, multiple genotypical H9N2 and H5N1 isolates were obtained recently from different terrestrial birds [16], [34], suggesting that these species may also play an important role in the evolution of influenza A virus. The findings that H9N2 reassortant viruses can be isolated from black-billed magpies indicated that black-billed magpies were susceptible to H9N2 viruses. Black-billed magpies are distributed throughout China and much of the world, and are closely associated with various kinds of poultry and wild birds, which raise concerns over possible transmissions of reassortant H9N2 viruses among poultry and wild birds.

In summary, our study is probably the first report to document and characterize H9N2 influenza viruses isolated from black-billed magpies in southern China. These BbM viruses were highly related to H5N1-like viruses in ribonucleoprotein complex genes, and are capable of replicating efficiently in chickens and mice without previous adaptation, suggesting that they may require relatively less genetic changes such as exchange of gene segments or accumulation of critical mutations to become more pathogenic to hosts or make the avian-to-mammalian transmission. Therefore, conducting long-term molecular epidemiological investigations of H9N2 influenza viruses in black-billed magpies is necessary to better understand the genetic evolution of BbM viruses and elucidate their potential role in future influenza outbreaks in poultry and epidemics in humans.

## Materials and Methods

### Ethics Statement

This study was approved by the Ethical Committee of Animal Experiments of the Institute of Zoology, Chinese Academy of Sciences, which does not issue a number to any animal study. All animal care and use were conducted in strict accordance with the Animal Research Committee guidelines of the Institute of Zoology, Chinese Academy of Sciences. All surgery was performed under anesthesia, and all efforts were made to minimize suffering.

### Isolation of avian influenza viruses

Two epidemic surveys on avian influenza viruses in wild black-billed magpies were conducted at the mountain forest near Zhongzhou village in Guangxi, China in March and May of 2005. Briefly, tracheal and cloacal swabs were collected from trapped black-billed magpies, and were eluted with PBS containing 2000U/ml penicillin and 1000ug/ml streptomycin at 4°C for 12 h. Samples were centrifuged at 5000 g for 10 min at 4°C and the supernatant was inoculated into the allantoic cavity of 10-day-old SPF embryonated chicken eggs. Inoculated embryos were incubated at 37°C and allantoic fluid was harvested 48–72 h p.i. [6], [34]. Subtypes of influenza viruses were determined by hemagglutination-inhibition (HI) and neuraminidase inhibition (NI) tests as previously described [39]. Virus stocks were stored at -70°C for further experiments.

### Sequencing of viral genes

Viral RNA was extracted from infected allantoic fluid with Trizol LS Reagent (Invitrogen) and was reverse-transcribed into cDNA by using universal influenza primer with the M-MLV reverse transcriptase (Promega). Eight full-length influenza virus genes were amplified by using the TaKaRa Ex Taq^TM^ DNA polymerase with specific primers (primer sequences available on request). The PCR products were purified with an agarose gel DNA extraction kit (Tiangen) and directly sequenced by the Invitrogen Biotechnology Company. Obtained sequences were assembled and compiled with the SeqMan program of the Lasergene 6.0 (DNASTAR, Madison, WI), and were deposited in GenBank.

### Phylogenetic and molecular analyses

To better understand the evolution of H9N2 viruses in black-billed magpies, the complete genomes of BbM viruses were sequenced and analyzed phylogenetically, and neighbor-joining phylogenetic trees were constructed for eight gene segments based on nucleotide sequences. The reference sequences were available from GenBank (Table S1). Nucleotide sequences of eight gene segments were aligned by Clustal W in the BioEdit version 7.0.5 program (http://www.mbio.ncsu.edu/BioEdit/bioedit.html). To determine sequence similarities and evolutionary relationships, pairwise sequence alignments were performed and neighbor-joining trees were constructed using the MEGA version 4.0.2 program (http://www.megasoftware.net/). Amino acid sequences of HA, NA, PB2, PB1, M and NS proteins were also aligned and analyzed by Clustal W in the BioEdit program. Potential N-linked glycosylation sites in HA and NA proteins were predicted with the NetNGlyc 1.0 server (http://www.cbs.dtu.dk/services/NetNGlyc/).

### Genotypical analysis

According to available genomic information of different H9N2 genotypes from our recent study [4], genotypic analysis was performed here. Genes sharing over 95% homology in the same lineage were considered as one genotypic group (22). H9N2 viruses with different gene constellations were divided according to their HA lineages into genotype A–G series. Genotypes of H9N2 viruses were determined and summarized in Table 1.

### Pathogenicity studies

To determine the pathogenicity of isolated viruses in chickens, 6-week-old, SPF White Leghorn chickens (Beijing MERIAL Ltd, 10 chickens/group) were i.n. inoculated with 0.1 ml 10^6.0^ EID_50_ of virus-allantoic fluid [22], and were observed for signs of disease and deaths over a period of 21 days. Control chickens were inoculated with PBS. On days 3 and 5 p.i., cloacal and tracheal swab samples were collected for virus titration in eggs as described [40].

To determine the pathogenicity of isolated viruses in mice, groups of eight 8-week-old SPF female BALB/c mice (Experimental Animal Center of Beijing) were i.n. inoculated under anesthesia with 10^6.0^ EID_50_ of the virus-allantoic fluid in a volume of 0.05 ml, and were observed daily for 14 days for signs of disease. Control mice were inoculated with PBS. On day 3, three mice from each group were sacrificed for collection of lungs, spleens, and brains under sterile conditions for virus titration in eggs [40]. Virus titers are given in units of log_10_EID_50_ per 1 ml ± standard deviation (SD). The remaining inoculated mice were monitored daily for weight loss and mortality.

### Antigenic analysis

Antigenic analysis was performed using antisera to three different H9N2 viruses generated in 6-week-old SPF White Leghorn chickens. The antigenicity of three BbM H9N2 influenza viruses were determined by HI assays as described previously [22].

### Nucleotide sequence accession numbers

The nucleotide sequences for influenza virus genes obtained from this study have been deposited to GenBank under accession numbers GU121379 to GU121386 and HM590759 to HM590774.

## Supporting Information

Figure S1 **Potential N-linked glycosylation sites in HA and NA proteins of the BbM viruses (BbM/GX/29/05 as a template) predicted by the NetNGlyc 1.0 Server analysis.**
(TIF)

Figure S2 **Potential N-linked glycosylation sites in HA1 proteins of the BbM viruses based on the 1jsdA HA1 template.** In blue HA1 protein, red arrow-head indicates mutable potential glycosylation sites at positions 82 and 218, while black arrow-head indicates conserved potential glycosylation sites at positions 29, 141, 298 and 305.
(TIF)

Table S1 **Accession numbers of nucleic acid sequences used in this study.**
(DOC)

Table S2 **Amino acid differences at the whole PB1 protein between the BbM viruses (BbM/GX/29/05 as a template) and analyzed reference viruses from different lineages.**
(DOC)
