# Supplementary figures and images for: Reassortant H9N2 Influenza Viruses Containing H5N1-Like PB1 Genes Isolated from Black-Billed Magpies in Southern China
Source: PLoS One. 2011 Sep 29;6(9):e25808. doi: 10.1371/journal.pone.0025808 (PMC3183077; doi:10.1371/journal.pone.0025808)

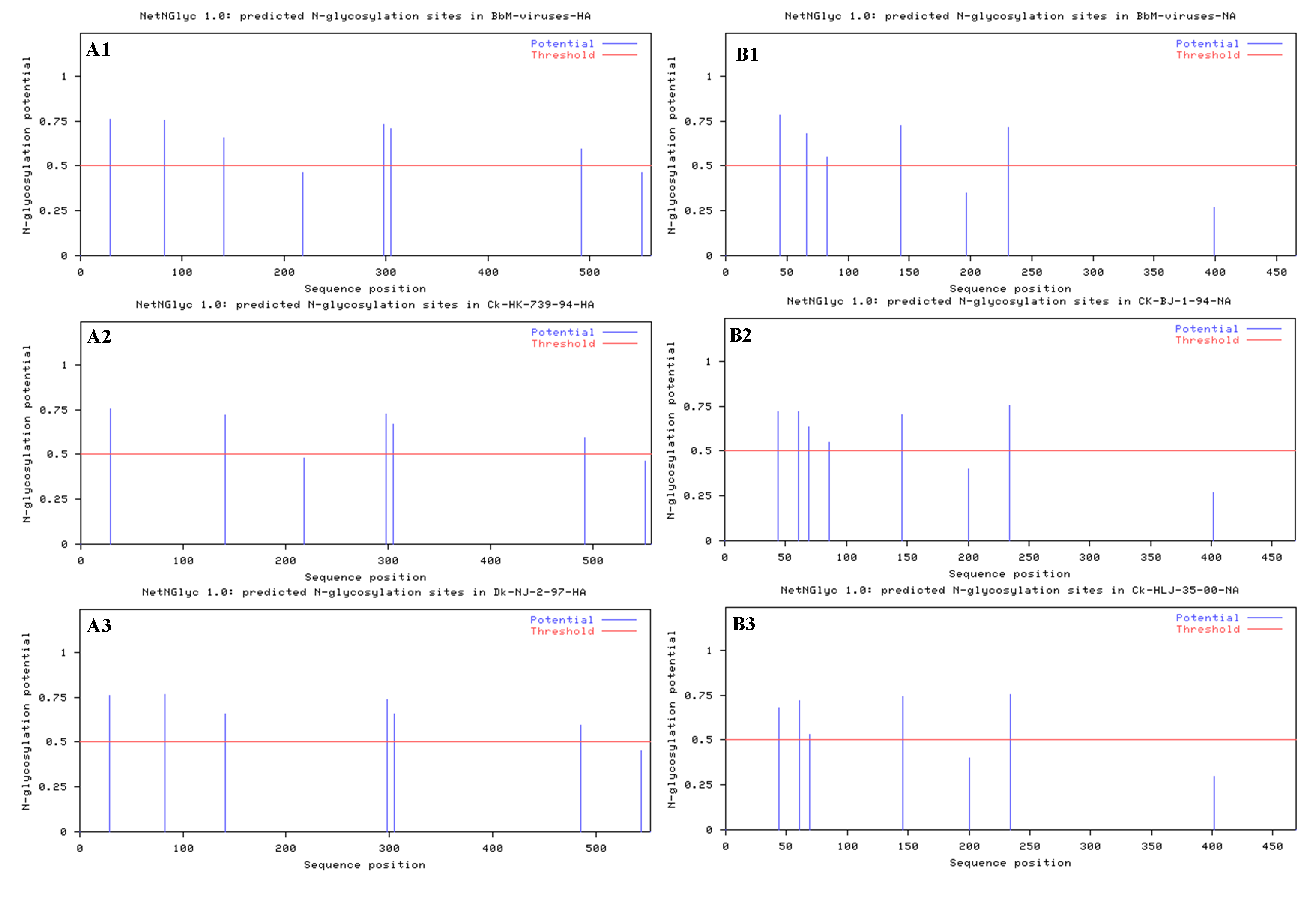

Supplement: Figure S1 — Potential N-linked glycosylation sites in HA and NA proteins of the BbM viruses (BbM/GX/29/05 as a template) predicted by the NetNGlyc 1.0 Server analysis. (TIF) [file pone.0025808.s001.tif]

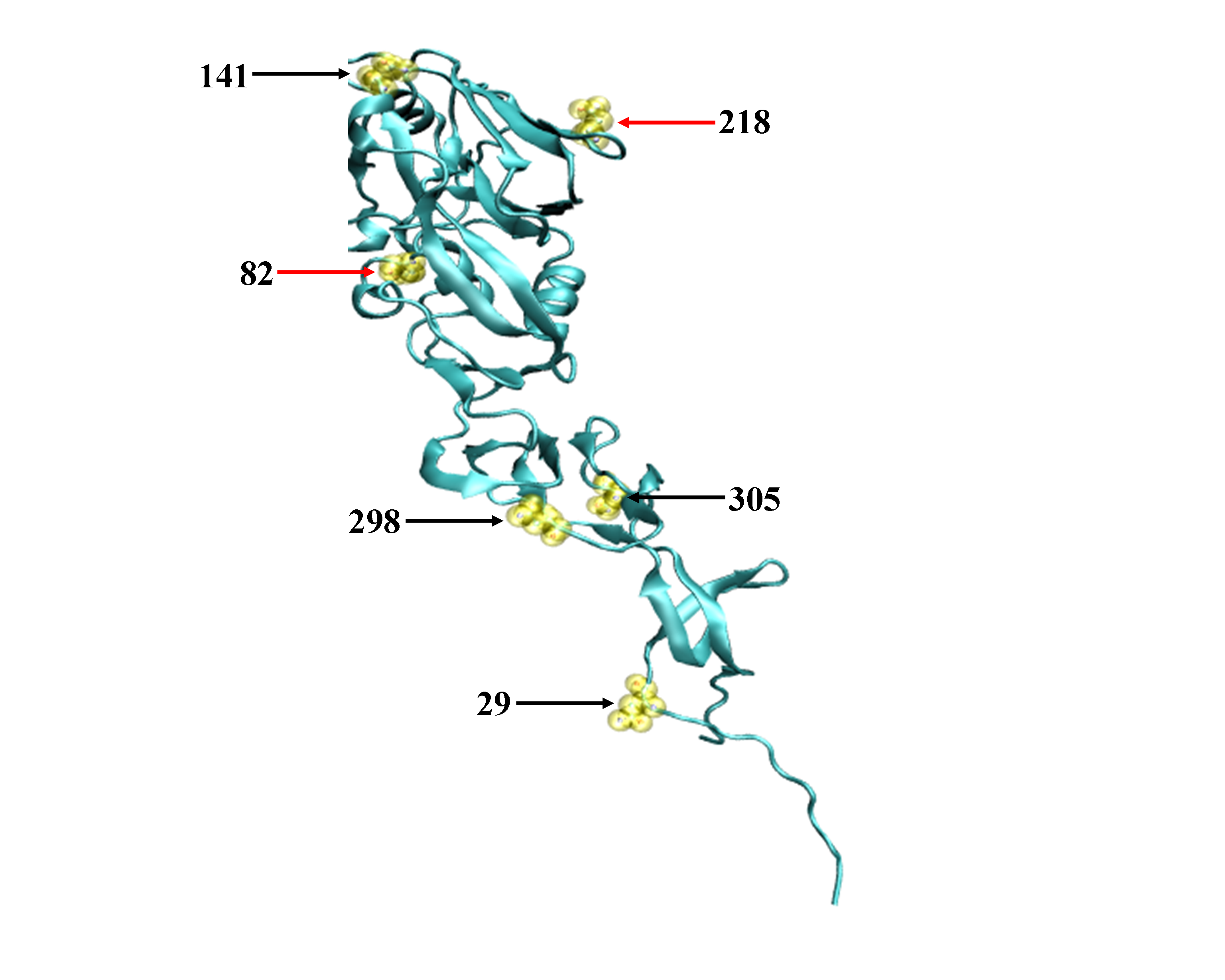

Supplement: Figure S2 — Potential N-linked glycosylation sites in HA1 proteins of the BbM viruses based on the 1jsdA HA1 template. In blue HA1 protein, red arrow-head indicates mutable potential glycosylation sites at positions 82 and 218, while black arrow-head indicates conserved potential glycosylation sites at positions 29, 141, 298 and 305. (TIF) [file pone.0025808.s002.tif]
