# Supplementary material for: Reassortant H9N2 Influenza Viruses Containing H5N1-Like PB1 Genes Isolated from Black-Billed Magpies in Southern China
Source: PLoS One. 2011 Sep 29;6(9):e25808. doi: 10.1371/journal.pone.0025808 (PMC3183077; doi:10.1371/journal.pone.0025808)
Supplement: Table S1 — Accession numbers of nucleic acid sequences used in this study. (DOC) [file pone.0025808.s003.doc]

| **PB2** | | **PB1** | | **PA** | | **HA** | | **NP** | | **NA** | | **MP** | | **NS** | |
| --- | --- | --- | --- | --- | --- | --- | --- | --- | --- | --- | --- | --- | --- | --- | --- |
| **Virus** | **Accession** | **Virus** | **Accession** | **Virus** | **Accession** | **Virus** | **Accession** | **Virus** | **Accession** | **Virus** | **Accession** | **Virus** | **Accession** | **Virus** | **Accession** |
| BbM/GX/29/05(H9N2) | GU121379 | BbM/GX/29/05(H9N2) | GU121380 | BbM/GX/29/05(H9N2) | GU121381 | BbM/GX/29/05(H9N2) | GU121382 | BbM/GX/29/05(H9N2) | GU121383 | BbM/GX/29/05(H9N2) | GU121384 | BbM/GX/29/05(H9N2) | GU121385 | BbM/GX/29/05(H9N2) | GU121386 |
| BbM/GX/30/05(H9N2) | HM590759 | BbM/GX/30/05(H9N2) | HM590760 | BbM/GX/30/05(H9N2) | HM590761 | BbM/GX/30/05(H9N2) | HM590762 | BbM/GX/30/05(H9N2) | HM590763 | BbM/GX/30/05(H9N2) | HM590764 | BbM/GX/30/05(H9N2) | HM590765 | BbM/GX/30/05(H9N2) | HM590766 |
| BbM/GX/31/05(H9N2) | HM590767 | BbM/GX/31/05(H9N2) | HM590768 | BbM/GX/31/05(H9N2) | HM590769 | BbM/GX/31/05(H9N2) | HM590770 | BbM/GX/31/05(H9N2) | HM590771 | BbM/GX/31/05(H9N2) | HM590774 | BbM/GX/31/05(H9N2) | HM590772 | BbM/GX/31/05(H9N2) | HM590773 |
| Ck/BJ/1/94(H9N2) | AF156438 | Ck/BJ/1/94(H9N2) | AF156423 | Ck/BJ/1/94(H9N2) | AF156452 | Ck/BJ/1/94(H9N2) | AF156380 | Ck/BJ/1/94(H9N2) | AF156409 | Ck/BJ/1/94(H9N2) | AF156398 | Ck/BJ/1/94(H9N2) | AF156466 | Ck/BJ/1/94(H9N2) | AF156480 |
| Ck/HK/739/94(H9N2) | AF156436 | Ck/HK/739/94(H9N2) | AF156422 | Ck/HK/739/94(H9N2) | AF156450 | Ck/HK/739/94(H9N2) | AF156379 | Ck/HK/739/94(H9N2) | AF156408 | Ck/HK/739/94(H9N2) | AF156397 | Ck/HK/739/94(H9N2) | AF156464 | Ck/HK/739/94(H9N2) | AF156478 |
| DkK/DE/113/95(H9N2) | AF508644 | DkK/DE/113/95(H9N2) | AF508622 | DkK/DE/113/95(H9N2) | AF508666 | Ck/TJ/1/96(H9N2) | AF461526 | DkK/DE/113/95(H9N2) | AF508600 | DkK/DE/113/95(H9N2) | AF508578 | Ck/BJ/1/95(H9N2) | AF536719 | Ck/BJ/1/95(H9N2) | AF536729 |
| Ck/SD/6/96(H9N2) | DQ064565 | Ck/SD/6/96(H9N2) | DQ064538 | Ck/SD/6/96(H9N2) | DQ064511 | DkK/DE/113/95(H9N2) | AF218100 | Ck/SD/6/96(H9N2) | DQ064457 | Ck/KR/323/96(H9N2) | AF156400 | DkK/DE/113/95(H9N2) | AF508688 | DkK/DE/113/95(H9N2) | AF508709 |
| Ck/KR/006/96(H9N2) | AF156440 | Ck/KR/006/96(H9N2) | AF156426 | Ck/KR/006/96(H9N2) | AF156454 | Ck/SD/6/96(H9N2) | DQ064376 | Ck/KR/006/96(H9N2) | AF156412 | Ck/GD/6/97(H9N2) | DQ064416 | Ck/SD/6/96(H9N2) | DQ064403 | Ck/SD/6/96(H9N2) | DQ064484 |
| Ck/KR/323/96(H9N2) | AF156439 | Ck/KR/323/96(H9N2) | AF156425 | Ck/KR/323/96(H9N2) | AF156453 | Ck/KR/006/96(H9N2) | AF156385 | Ck/KR/323/96(H9N2) | AF156411 | Dk/NJ/2/97(H9N2) | DQ064428 | Ck/KR/006/96(H9N2) | AF156468 | Ck/KR/006/96(H9N2) | AF156482 |
| Ck/GD/6/97(H9N2) | DQ064551 | Ck/GD/6/97(H9N2) | DQ064524 | Ck/GD/6/97(H9N2) | DQ064497 | Ck/KR/323/96(H9N2) | AF156384 | Ck/GD/6/97(H9N2) | DQ064443 | Ck/HK/G23/97(H9N2) | AF156392 | Ck/KR/323/96(H9N2) | AF156467 | Ck/KR/323/96(H9N2) | AF156481 |
| Dk/NJ/2/97(H9N2) | DQ064563 | Dk/NJ/2/97(H9N2) | DQ064536 | Dk/NJ/2/97(H9N2) | DQ064509 | Ck/GD/6/97(H9N2) | DQ064362 | Dk/NJ/2/97(H9N2) | DQ064455 | Ck/HK/G9/97(H9N2) | AF156391 | Ck/GD/6/97(H9N2) | DQ064389 | Ck/GD/6/97(H9N2) | DQ064470 |
| Ck/HK/G23/97(H9N2) | AF156431 | Ck/HK/G23/97(H9N2) | AF156417 | Ck/HK/G23/97(H9N2) | AF156445 | Dk/NJ/2/97(H9N2) | DQ064374 | Ck/HK/G9/97(H9N2) | AF156402 | Pg/HK/Y233/97(H9N2) | AF156393 | Dk/NJ/2/97(H9N2) | DQ064401 | Dk/NJ/2/97(H9N2) | DQ064482 |
| Ck/HK/G9/97(H9N2) | AF156430 | Ck/HK/G9/97(H9N2) | AF156416 | Ck/HK/G9/97(H9N2) | AF156444 | Ck/HK/G23/97(H9N2) | AF156374 | Pg/HK/Y233/97(H9N2) | AF156404 | Qa/HK/G1/97(H9N2) | AF156396 | Ck/HK/G9/97(H9N2) | AF156458 | Ck/HK/G9/97(H9N2) | AF156472 |
| Pg/HK/Y233/97(H9N2) | AF156432 | Pg/HK/Y233/97(H9N2) | AF156418 | Pg/HK/Y233/97(H9N2) | AF156446 | Ck/HK/G9/97(H9N2) | AF156373 | Qa/HK/G1/97(H9N2) | AF156407 | Dk/HK/Y280/97(H9N2) | AF156394 | Qa/HK/G1/97(H9N2) | AF156463 | Qa/HK/G1/97(H9N2) | AF156477 |
| Qa/HK/G1/97(H9N2) | AF156435 | Qa/HK/G1/97(H9N2) | AF156421 | Qa/HK/G1/97(H9N2) | AF156449 | Qa/HK/G1/97(H9N2) | AF156378 | Dk/HK/Y280/97(H9N2) | AF156407 | Dk/HK/Y439/97(H9N2) | AF156395 | Dk/HK/Y280/97(H9N2) | AF156461 | Dk/HK/Y280/97(H9N2) | AF156475 |
| Dk/HK/Y280/97(H9N2) | AF156433 | Dk/HK/Y280/97(H9N2) | AF156419 | Dk/HK/Y280/97(H9N2) | AF156447 | Dk/HK/Y280/97(H9N2) | AF156376 | Dk/HK/Y439/97(H9N2) | AF156406 | Ph/IE/PV18/97(H9N2) | AF508581 | Dk/HK/Y439/97(H9N2) | AF156462 | Dk/HK/Y439/97(H9N2) | AF156476 |
| Dk/HK/Y439/97(H9N2) | AF156434 | Dk/HK/Y439/97(H9N2) | AF156420 | Dk/HK/Y439/97(H9N2) | AF156448 | Dk/HK/Y439/97(H9N2) | AF156377 | Ph/IE/PV18/97(H9N2) | AF508603 | Pa/Chiba/1/97(H9N2) | AB049163 | Ph/IE/PV18/97(H9N2) | AF508691 | Ph/IE/PV18/97(H9N2) | AF508712 |
| Ph/IE/PV18/97(H9N2) | AF508647 | Ph/IE/PV18/97(H9N2) | AF508625 | Ph/IE/PV18/97(H9N2) | AF156448 | Pa/Chiba/1/97(H9N2) | AB049159 | Pa/Chiba/1/97(H9N2) | AB049161 | Ck/SH/F/98(H9N2) | AY253754 | Pa/Chiba/1/97(H9N2) | AB049165 | Pa/Chiba/1/97(H9N2) | AB049167 |
| Pa/Chiba/1/97(H9N2) | AB049153 | Pa/Chiba/1/97(H9N2) | AB049155 | Pa/Chiba/1/97(H9N2) | AB049157 | Ck/SH/F/98(H9N2) | AY743216 | Ck/SH/F/98(H9N2) | AY253753 | Ck/BJ/8/98(H9N2) | DQ064434 | Ck/SH/F/98(H9N2) | AY253755 | Ck/SH/F/98(H9N2) | AY253756 |
| Ck/SH/F/98(H9N2) | AY253750 | Ck/SH/F/98(H9N2) | AY253751 | Ck/SH/F/98(H9N2) | AY253752 | Ck/BJ/8/98(H9N2) | DQ064354 | Ck/BJ/8/98(H9N2) | DQ064461 | Pa/Narita/92A/98(H9N2) | AB049164 | Ck/BJ/8/98(H9N2) | DQ064407 | Ck/BJ/8/98(H9N2) | AF508714 |
| Ck/BJ/8/98(H9N2) | DQ064543 | Ck/BJ/8/98(H9N2) | DQ064542 | Ck/BJ/8/98(H9N2) | DQ064515 | Pa/Narita/92A/98(H9N2) | AB049160 | Ck/GX/10/99(H9N2) | DQ064444 | Ck/GX/9/99(H9N2) | DQ064418 | Ck/GX/10/99(H9N2) | DQ064390 | Pa/Narita/92A/98(H9N2) | AB049168 |
| Ck/DE/R45/98(H9N2) | AF508643 | Pa/Narita/92A/98(H9N2) | AB049156 | Pa/Narita/92A/98(H9N2) | AB049158 | Ck/GX/10/99(H9N2) | DQ064363 | Ck/GX/9/99(H9N2) | DQ064445 | Dk/ST/1796/00(H9N2) | AF523401 | Ck/GX/9/99(H9N2) | DQ064391 | Ck/GX/10/99(H9N2) | DQ064471 |
| Pa/Narita/92A/98(H9N2) | AB049154 | Ck/GX/10/99(H9N2) | DQ064525 | Ck/GX/10/99(H9N2) | DQ064498 | Ck/GX/9/99(H9N2) | DQ064364 | Ck/Pk/2/99(H9N2) | AJ291394 | Dk/ST/2134/00(H9N2) | AF523393 | Ck/KR/99029/99(H9N2) | AF508692 | Ck/GX/9/99(H9N2) | DQ064472 |
| Ck/GX/10/99(H9N2) | DQ064552 | Ck/GX/9/99(H9N2) | DQ064526 | Ck/GX/9/99(H9N2) | DQ064499 | Ck/Pk/2/99(H9N2) | AJ291392 | Ck/KR/99029/99(H9N2) | AF508604 | Dk/ST/830/00(H9N2) | AF523400 | Ck/FJ/25/00(H9N2) | DQ064382 | Dk/ST/1796/00(H9N2) | AF523509 |
| Ck/GX/9/99(H9N2) | DQ064553 | Ck/Pk/2/99(H9N2) | AJ291396 | Ck/Pk/2/99(H9N2) | AJ291397 | Cken/LN/1/00(H9N2) | AF461518 | Ck/FJ/25/00(H9N2) | DQ064436 | Ck/KR/99029/99(H9N2) | AF508582 | Ck/GD/4/00(H9N2) | DQ064385 | Dk/ST/830/00(H9N2) | AF523512 |
| Ck/Pk/2/99(H9N2) | AJ291395 | Ck/KR/99029/99(H9N2) | AF508626 | Ck/KR/99029/99(H9N2) | AF508670 | Dk/ST/1796/00(H9N2) | AF523375 | Ck/GD/4/00(H9N2) | DQ064439 | Ck/FJ/25/00(H9N2) | DQ064409 | Ck/HLJ/35/00(H9N2) | DQ064393 | Ck/KR/99029/99(H9N2) | AF508713 |
| Ck/KR/99029/99(H9N2) | AF508648 | Ck/FJ/25/00(H9N2) | DQ064517 | Ck/FJ/25/00(H9N2) | DQ064490 | Dk/ST/2134/00(H9N2) | AF523380 | Ck/HLJ/35/00(H9N2) | DQ064447 | Ck/GD/4/00(H9N2) | DQ064412 | Qa/ST/243/00(H9N2) | EF154980 | Ck/FJ/25/00(H9N2) | DQ064463 |
| Ck/FJ/25/00(H9N2) | DQ064544 | Ck/GD/4/00(H9N2) | DQ064520 | Ck/GD/4/00(H9N2) | DQ064493 | Ck/FJ/25/00(H9N2) | DQ064355 | Qa/ST/243/00(H9N2) | EF155126 | Ck/HLJ/35/00(H9N2) | DQ064420 | Qa/ST/2816/00(H9N2) | EF154986 | Ck/GD/4/00(H9N2) | DQ064466 |
| Ck/GD/4/00(H9N2) | DQ064548 | Ck/HLJ/35/00(H9N2) | DQ064528 | Ck/HLJ/35/00(H9N2) | DQ064501 | Ck/GD/4/00(H9N2) | DQ064358 | Qa/ST/2816/00(H9N2) | EF155132 | Qa/ST/243/00(H9N2) | EF155053 | KR/0028/00(H9N2) | EF620903 | Ck/HLJ/35/00(H9N2) | DQ064474 |
| Ck/HLJ/35/00(H9N2) | DQ064555 | KR/0028/00(H9N2) | EF620898 | Qa/ST/243/00(H9N2) | EF155272 | Ck/HLJ/35/00(H9N2) | DQ064366 | KR/0028/00(H9N2) | EF620901 | KR/0028/00(H9N2) | EF620902 | Qa/NC/2-0460/00(H9N2) | CY005501 | Qa/ST/243/00(H9N2) | EF155199 |
| KR/0028/00(H9N2) | EF620897 | Pg/NC/2-0461/00(H9N2) | CY005511 | KR/0028/00(H9N2) | EF620899 | Qa/ST/1820/00(H9N2) | EF154911 | Pg/NC/2-0461/00(H9N2) | CY005509 | Pg/NC/2-0461/00(H9N2) | CY005508 | WD/NC/2-0480/00(H9N2) | CY005513 | KR/0028/00(H9N2) | EF620904 |
| Pg/NC/2-0461/00(H9N2) | CY005512 | Qa/NC/2-0460/00(H9N2) | CY005505 | Pg/NC/2-0461/00(H9N2) | CY005510 | KR/0028/00(H9N2) | EF620900 | Qa/NC/2-0460/00(H9N2) | CY005503 | Qa/NC/2-0460/00(H9N2) | CY005502 | Dk/ST/1605/01(H9N2) | AF523493 | Qa/NC/2-0460/00(H9N2) | CY006019 |
| Qa/NC/2-0460/00(H9N2) | CY005506 | WD/NC/2-0480/00(H9N2) | CY005517 | Qa/NC/2-0460/00(H9N2) | CY005504 | Pg/NC/2-0461/00(H9N2) | CY014613 | WD/NC/2-0480/00(H9N2) | CY005515 | WD/NC/2-0480/00(H9N2) | CY005514 | Ck/GD/56/01(H9N2) | DQ064388 | WD/NC/2-0480/00(H9N2) | CY006022 |
| Ck/GD/56/01(H9N2) | DQ064550 | Ck/HLJ/48/01(H9N2) | DQ064529 | WD/NC/2-0480/00(H9N2) | CY005516 | Qa/NC/2-0460/00(H9N2) | CY006018 | Ck/GD/56/01(H9N2) | DQ064442 | Dk/ST/1605/01(H9N2) | AF523402 | Ck/HLJ/48/01(H9N2) | DQ064394 | Dk/ST/1605/01(H9N2) | AF523504 |
| Ck/HLJ/48/01(H9N2) | DQ064556 | Ck/SH/10/01(H9N2) | DQ064540 | Ck/GD/56/01(H9N2) | DQ064496 | Ck/GD/56/01(H9N2) | DQ064361 | Ck/HLJ/48/01(H9N2) | DQ064448 | Gf/ST/2076/01(H9N2) | CY024562 | Ck/SH/10/01(H9N2) | DQ064405 | Ck/GD/56/01(H9N2) | DQ064469 |
| Ck/SH/10/01(H9N2) | DQ064567 | Ck/HeN/43/02(H9N2) | DQ064531 | Ck/HLJ/48/01(H9N2) | DQ064502 | Ck/HLJ/48/01(H9N2) | DQ064367 | Ck/SH/10/01(H9N2) | DQ064459 | Ck/GD/56/01(H9N2) | DQ064415 | WD/ST/4808/01(H9N2) | AF523484 | Ck/HLJ/48/01(H9N2) | DQ064475 |
| Ck/HeN/43/02(H9N2) | DQ064558 | Ty/CA/189/66(H9N2) | AF156429 | Ck/SH/10/01(H9N2) | DQ064513 | Ck/SH/10/01(H9N2) | DQ064378 | Qu/ST/1425/01(H9N2) | EF155138 | Ck/HLJ/48/01(H9N2) | DQ064421 | Ck/HeN/43/02(H9N2) | DQ064396 | Ck/SH/10/01(H9N2) | DQ064486 |
| Ck/KR/S1/03(H9N2) | AY800240 | Ty/WI/1/66(H9N2) | CY014669 | Qa/ST/1242/01(H9N2) | EF155283 | Qa/ST/1158/01(H9N2) | EF154916 | Ck/HeN/43/02(H9N2) | DQ064450 | Ck/SH/10/01(H9N2) | DQ064432 | Qa/ST/3851/02(H9N2) | EF155010 | WD/ST/4808/01(H9N2) | AF523514 |
| Ty/CA/189/66(H9N2) | AF156443 | HK/1073/99(H9N2) | AF258816 | Qu/ST/1425/01(H9N2) | EF155284 | Ck/KR/01310/01(H9N2) | EU253561 | Ph/HK/WF54/03(H9N2) | AY664730 | Qa/ST/1242/01(H9N2) | EF155064 | Ph/HK/WF54/03(H9N2) | AY664692 | Ck/HeN/43/02(H9N2) | DQ064477 |
| Ty/WI/1/66(H9N2) | CY014670 | HK/1074/99(H9N2) | AF258817 | Ck/HeN/43/02(H9N2) | DQ064504 | Ck/HeN/43/02(H9N2) | DQ064369 | Dk/KR/S13/03(H9N2) | AY862650 | Qu/ST/1425/01(H9N2) | EF155065 | Dk/KR/S13/03(H9N2) | AY862618 | Ph/HK/WF54/03(H9N2) | AY664749 |
| HK/1073/99(H9N2) | AF258835 | GZ/333/99(H9N2) | AY043029 | Ty/CA/189/66(H9N2) | AF156457 | Qa/ST/1038/02(H9N2) | EF154935 | Ty/CA/189/66(H9N2) | AF156415 | WD/ST/4808/01(H9N2) | AF523394 | Chu/ST/22116/05(H9N2) | CY024555 | Dk/KR/S13/03(H9N2) | AY862666 |
| HK/1074/99(H9N2) | AF258836 | HK/2108/03(H9N2) | DQ226161 | Ty/WI/1/66(H9N2) | CY014668 | Ck/HK/FY23/03(H9N2) | AY664665 | Ty/WI/1/66(H9N2) | CY014666 | Ck/HeN/43/02(H9N2) | DQ064423 | Ty/CA/189/66(H9N2) | AF156471 | Qa/ST/403/04(H9N2) | EF155250 |
| HK/2108/03(H9N2) | DQ226172 | Bird/GX/62/05(H9N2) | EU086279 | HK/1073/99(H9N2) | AF257191 | Gf/HK/NT184/03(H9N2) | AY664674 | HK/1073/99(H9N2) | AF255742 | Qa/ST/1038/02(H9N2) | EF155081 | Ty/WI/1/66(H9N2) | CY014664 | Chu/ST/22116/05(H9N2) | CY024556 |
| Bird/GX/62/05(H9N2) | EU086281 | Qa/GX/B1/06(H9N2) | EU086297 | HK/1074/99(H9N2) | AF257192 | Ph/HK/WF54/03(H9N2) | AY664673 | HK/1074/99(H9N2) | AF255743 | Qa/ST/3502/03(H9N2) | EF155096 | HK/1073/99(H9N2) | AF255363 | Ty/CA/189/66(H9N2) | AF156485 |
| Qa/GX/B1/06(H9N2) | EU086299 | Sw/GX/FS2/05(H9N2) | EU086314 | GZ/333/99(H9N2) | AY043028 | Ck/KR/S18/03(H9N2) | AY862606 | Qa/HK/A17/99(H9N2) | AF222614 | Ck/HK/FY23/03(H9N2) | AY664703 | HK/1074/99(H9N2) | AF255364 | Ty/WI/1/66(H9N2) | CY014667 |
| Sw/GX/FS2/05(H9N2) | EU086316 | Bird/GX/H1/06(H9N2) | EU086315 | Bird/GX/62/05(H9N2) | EU086257 | Qa/ST/6648/04(H9N2) | EF154964 | HK/2108/03(H9N2) | DQ226139 | Gf/HK/NT184/03(H9N2) | AY664712 | Qa/HK/A17/99(H9N2) | AF222662 | HK/1073/99(H9N2) | AJ278649 |
| Bird/GX/H1/06(H9N2) | EU086317 | Sw/GX/S11/05(H9N2) | EU086330 | Qa/GX/B1/06(H9N2) | EU086294 | Chu/ST/22116/05(H9N2) | CY024552 | Bird/GX/62/05(H9N2) | EU086252 | Ph/HK/WF54/03(H9N2) | AY664711 | HK/2108/03(H9N2) | DQ226095 | HK/1074/99(H9N2) | AF256177 |
| Sw/GX/S11/05(H9N2) | EU086332 | Sw/GX/S15/05(H9N2) | EU086331 | Sw/GX/FS2/05(H9N2) | EU086313 | Qa/ST/15892/05(H9N2) | EF154976 | Qa/GX/B1/06(H9N2) | EU086289 | Pg/HK/WF53/03(H9N2) | AY664710 | Bird/GX/62/05(H9N2) | EU086247 | Qa/HK/A17/99(H9N2) | AF222672 |
| Sw/GX/S15/05(H9N2) | EU086333 | Gs/GD/1/96(H5N1) | AF144301 | Bird/GX/H1/06(H9N2) | EU086312 | Ty/DE/R22/96(H9N2) | AJ781820 | Sw/GX/FS2/05(H9N2) | EU086309 | Dk/KR/S13/03(H9N2) | AY862634 | Qa/GX/B1/06(H9N2) | EU086285 | Bird/GX/62/05(H9N2) | EU086255 |
| Gs/GD/1/96(H5N1) | AF144300 | Dk/SH/35/02(H5N1) | AY585499 | Sw/GX/S11/05(H9N2) | EU086329 | Ty/DE/R33/96(H9N2) | AJ781821 | Bird/GX/H1/06(H9N2) | EU086308 | Qa/ST/6046/04(H9N2) | EF155109 | Sw/GX/FS2/05(H9N2) | EU086304 | Qa/GX/B1/06(H9N2) | EU086292 |
| Dk/SH/35/02(H5N1) | AY585520 | Ck/HK/YU22/02(H5N1) | AY651680 | Sw/GX/S15/05(H9N2) | EU086328 | Ty/CA/189/66(H9N2) | AF156390 | Sw/GX/S11/05(H9N2) | EU086325 | Chu/ST/22116/05(H9N2) | CY024554 | Bird/GX/H1/06(H9N2) | EU086305 | Sw/GX/FS2/05(H9N2) | EU086310 |
| Ck/HK/YU22/02(H5N1) | AY651734 | Ck/YN/1252/03(H5N1) | CY029390 | Gs/GD/1/96(H5N1) | AF144302 | Ty/WI/1/66(H9N2) | DQ067444 | Sw/GX/S15/05(H9N2) | EU086324 | Qa/ST/15892/05(H9N2) | EF155122 | Sw/GX/S11/05(H9N2) | EU086320 | Bird/GX/H1/06(H9N2) | EU086311 |
| Ck/YN/1252/03(H5N1) | CY029389 | Ck/HeN/01/04(H5N1) | CY029390 | Dk/SH/35/02(H5N1) | AY585478 | HK/1073/99(H9N2) | AJ404626 | Gs/GD/1/96(H5N1) | AF144303 | Ty/CA/189/66(H9N2) | AF156401 | Sw/GX/S15/05(H9N2) | EU086321 | Sw/GX/S11/05(H9N2) | EU08632 |
| Ck/HeN/01/04(H5N1) | AY950279 | DK/NC/8-174/00(H3N6) | CY005467 | Ck/HK/YU22/02(H5N1) | AY651626 | HK/1074/99(H9N2) | AJ404627 | Dk/SH/35/02(H5N1) | AY585436 | Ty/WI/1/66(H9N2) | CY014665 | Gs/GD/1/96(H5N1) | AF144306 | Sw/GX/S15/05(H9N2) | EU086326 |
| DK/NC/8-174/00(H3N6) | CY005468 | Bird/GX/A1/06(H9N2) | EU086298 | Ck/YN/1252/03(H5N1) | CY029391 | Bird/GX/62/05(H9N2) | EU086246 | Ck/HK/YU22/02(H5N1) | AY651514 | HK/1073/99(H9N2) | AJ404629 | Dk/SH/35/02(H5N1) | AY585393 | Gs/GD/1/96(H5N1) | AF144307 |
| Bird/GX/A1/06(H9N2) | EU08630 | Ck/HB/L1/06(H9N2) | EU882861 | Ck/HeN/01/04(H5N1) | AY950265 | Qa/GX/B1/06(H9N2) | EU086283 | Ck/YN/1252/03(H5N1) | CY029392 | HK/1074/99(H9N2) | AJ404628 | Ck/HK/YU22/02(H5N1) | AY651403 | Dk/SH/35/02(H5N1) | AY585457 |
| Ck/HB/L1/06(H9N2) | EU914201 | Ck/HB/C1/07(H9N2) | EU365369 | Bird/GX/A1/06(H9N2) | EU086295 | Sw/GX/FS2/05(H9N2) | EU086246 | Ck/HeN/01/04(H5N1) | AY950251 | Pg/HK/FY6/99(H9N2) | AF222653 | Ck/YN/1252/03(H5N1) | CY029394 | Ck/HK/YU22/02(H5N1) | AY651568 |
| Ck/HB/C1/07(H9N2) | EU365368 | Sw/GX/10/07(H9N2) | CY075052 | Ck/HB/L1/06(H9N2) | EU532061 | Bird/GX/H1/06(H9N2) | EU086303 | DK/NC/8-174/00(H3N6) | AY180547 | Qa/HK/A17/99(H9N2) | AF222652 | Ck/HeN/01/04(H5N1) | AY950237 | Ck/YN/1252/03(H5N1) | CY029395 |
| Sw/GX/10/07(H9N2) | CY075051 | Ck/SD/B2/07(H9N2) | EU939157 | Ck/HB/C1/07(H9N2) | EU365370 | Sw/GX/S11/05(H9N2) | EU086319 | Ck/HB/L1/06(H9N2) | EU532044 | HK/2108/03(H9N2) | DQ226128 | DK/NC/8-174/00(H3N6) | CY005463 | Ck/HeN/01/04(H5N1) | AY950258 |
| Ck/SD/B2/07(H9N2) | EU914194 | Sw/GX/7/07(H9N2) | CY075028 | Sw/GX/10/07(H9N2) | CY075053 | Sw/GX/S15/05(H9N2) | EU086318 | Ck/HB/C1/07(H9N2) | EU365372 | Bird/GX/62/05(H9N2) | EU086249 | CK/NC/3-120/01(H3N2) | CY005445 | DK/NC/8-174/00(H3N6) | CY005466 |
| Sw/GX/7/07(H9N2) | CY075027 | Sw/GX/8/07(H9N2) | CY075036 | Ck/SD/B2/07(H9N2) | EU532062 | Ck/HB/C1/07(H9N2) | EU365371 | Ck/SD/B2/07(H9N2) | EU414523 | Qa/GX/B1/06(H9N2) | EU086287 | Bird/GX/A1/06(H9N2) | EU086286 | Bird/GX/A1/06(H9N2) | EU086291 |
| Sw/GX/8/07(H9N2) | CY075035 | Sw/GX/9/07(H9N2) | CY075044 | Sw/GX/7/07(H9N2) | CY075029 | Ck/GX/17/07(H9N2) | GU722359 | Sw/GX/10/07(H9N2) | CY075055 | Sw/GX/FS2/05(H9N2) | EU086306 | Ck/HB/L1/06(H9N2) | EU532036 | Ck/HB/L1/06(H9N2) | EU532055 |
| Sw/GX/9/07(H9N2) | CY075043 | CK/HB/4/08(H9N2) | FJ499467 | Sw/GX/8/07(H9N2) | CY075037 | Sp/GX/121/07(H9N2) | GU722369 | Sw/GX/8/07(H9N2) | CY075039 | Bird/GX/H1/06(H9N2) | EU086307 | Ck/HB/C1/07(H9N2) | EU365374 | Ck/HB/C1/07(H9N2) | EU365375 |
| CK/HB/4/08(H9N2) | FJ499468 | Ck/HB/7/08(H9N2) | GQ202052 | Sw/GX/9/07(H9N2) | CY075045 | Ck/GX/G8/09(H9N2) | GU471805 | Sw/GX/9/07(H9N2) | CY075047 | Sw/GX/S11/05(H9N2) | EU086323 | Ck/SD/B2/07(H9N2) | EU414522 | Ck/SD/B2/07(H9N2) | EU532045 |
| Ck/HB/7/08(H9N2) | GQ202053 | Ck/HeN/L2/08(H9N2) | FJ492968 | CK/HB/4/08(H9N2) | FJ499470 | Dk/GD/810/09(H9N2) | GU471887 | CK/HB/4/08(H9N2) | FJ499469 | Ck/HB/C1/07(H9N2) | EU365373 | Sw/GX/10/07(H9N2) | CY075057 | Sw/GX/10/07(H9N2) | CY075058 |
| Ck/HeN/L2/08(H9N2) | EU935070 | Ck/SD/SG1/09(H9N2) | HM751184 | Ck/HB/7/08(H9N2) | GQ202055 | Ck/SD/C/09(H9N2) | HQ378727 | Ck/HB/7/08(H9N2) | GQ202054 | Ck/GX/17/07(H9N2) | GU72237 | Sw/GX/8/07(H9N2) | CY075041 | Sw/GX/7/07(H9N2) | CY075034 |
| Ck/SD/SG2/09(H9N2) | HM751191 | Ck/TB/S1/09(H9N2) | CY08716 | Ck/SD/SG1/09(H9N2) | HM751185 | Ck/JS/U1/10(H9N2) | JF715002 | Ck/HeN/L2/08(H9N2) | FJ492973 | Sp/GX/121/07(H9N2) | GU722380 | Sw/GX/9/07(H9N2) | CY075049 | CK/HB/4/08(H9N2) | FJ499465 |
| Ck/SD/SG1/09(H9N2) | HM751183 | Ck/TB/S4/09(H9N2) | CY087177 | Ck/SD/SG2/09(H9N2) | HM751193 | Ck/AH/LJT/10(H9N2) | JF715010 | Ck/SD/SG2/09(H9N2) | HM75119 | Ck/SD/1/08(H9N2) | HQ326723 | Sw/GX/7/07(H9N2) | CY075033 | Ck/HB/7/08(H9N2) | GQ202050 |
| Ck/TB/S4/09(H9N2) | Y087176 | Dk/TB/S2/09(H9N2) | CY087185 | Ck/TB/S1/09(H9N2) | CY087170 | Ck/SD/1/08(H9N2) | HQ326722 | Ck/TB/S1/09(H9N2) | CY087172 | Ck/SD/C/09(H9N2) | HQ378728 | CK/HB/4/08(H9N2) | J499466 | Ck/HeN/L2/08(H9N2) | EU835745 |
| Ck/TB/S1/09(H9N2) | CY087168 | Ck/AH10-01/10(H9N2) | F906204 | Ck/TB/S4/09(H9N2) | CY087178 | Ck/AH/AH16/08(H9N2) | FJ434579 | Ck/TB/S4/09(H9N2) | CY087180 | Sw/HB/012/08(H9N2) | CY063664 | Ck/HB/7/08(H9N2) | GQ202051 | Ck/SD/SG1/09(H9N2) | HM751190 |
| Dk/TB/S2/09(H9N2) | CY087184 |  |  | Dk/TB/S2/09(H9N2) | CY087186 | Ck/SD/22/08(H9N2) | GQ373073 | Dk/TB/S2/09(H9N2) | CY087188 | Ck/HB/LC/08(H9N2) | GQ373111 | Ck/HeN/L2/08(H9N2) | EU835749 | Ck/TB/S1/09(H9N2) | CY087175 |
| Ck/AH10-01/10(H9N2) | JF906203 |  |  | Ck/AH10-01/10(H9N2) | F906205 |  |  | Ck/AH10-01/10(H9N2) | JF906207 | Ck/SD/WL/09(H9N2) | HM751172 | Ck/SD/SG1/09(H9N2) | HM751189 | Dk/TB/S2/09(H9N2) | CY087191 |
|  |  |  |  |  |  |  |  |  |  | Sw/GX/S15/05(H9N2) | EU086322 | Ck/SD/SG2/09(H9N2) | HM751197 | Ck/AH10-01/10(H9N2) | JF906210 |
|  |  |  |  |  |  |  |  |  |  |  |  | Ck/TB/S1/09(H9N2) | CY087174 |  |  |
|  |  |  |  |  |  |  |  |  |  |  |  | Ck/TB/S4/09(H9N2) | CY087182 |  |  |
|  |  |  |  |  |  |  |  |  |  |  |  | Dk/TB/S2/09(H9N2) | CY087190 |  |  |
|  |  |  |  |  |  |  |  |  |  |  |  | Ck/AH10-01/10(H9N2) | JF906209 |  |  |
