# Supplementary material for: Reassortant H9N2 Influenza Viruses Containing H5N1-Like PB1 Genes Isolated from Black-Billed Magpies in Southern China
Source: PLoS One. 2011 Sep 29;6(9):e25808. doi: 10.1371/journal.pone.0025808 (PMC3183077; doi:10.1371/journal.pone.0025808)
Supplement: Table S2 — Amino acid differences at the whole PB1 protein between the BbM viruses (BbM/GX/29/05 as a template) and analyzed reference viruses from different lineages. (DOC) [file pone.0025808.s004.doc]

**Table.S2. Amino acid differences at the whole PB1 protein between the BbM viruses (BbM/GX/29/05 as a template) and analyzed reference viruses from different lineages.**

| **virus** | **lineage** | | **Amino acid residues at different positions in the encoding regions of the PB1 protein** | | | | | | | | | | | | | | | | | | | | | | | | | | | | | | | | | | | | | | | | | | | | | |  |
| --- | --- | --- | --- | --- | --- | --- | --- | --- | --- | --- | --- | --- | --- | --- | --- | --- | --- | --- | --- | --- | --- | --- | --- | --- | --- | --- | --- | --- | --- | --- | --- | --- | --- | --- | --- | --- | --- | --- | --- | --- | --- | --- | --- | --- | --- | --- | --- | --- | --- |
|  |  |  | **2** | | **3** | | **6** | | **14** | | **18** | | **23** | | **27** | | **38** | | **40** | | **42** | | **50** | | **53** | | **54** | | **59** | | **62** | | **64** | | **75** | | **76** | | **78** | | **102** | | **105** | | **111** | | **113** | |  |
| **BbM/GX/29/05(H9N2)** | | **H5N1-like** | | **D** | | **V** | | **T** | | **V** | | **I** | | **P** | | **D** | | **Y** | | **M** | | **T** | | **S** | | **G** | | **k** | | **T** | | **G** | | **P** | | **E** | | **D** | | **E** | | **I** | | **N** | | **M** | | **I** | |
| **BbM/GX/30/05(H9N2)** | | **H5N1-like** | |  | |  | |  | |  | |  | |  | |  | |  | |  | |  | |  | |  | |  | |  | |  | |  | |  | |  | |  | |  | |  | |  | |  | |
| **BbM/GX/31/05(H9N2)** | | **H5N1-like** | |  | |  | |  | |  | |  | |  | |  | |  | |  | |  | |  | |  | |  | |  | |  | |  | |  | |  | |  | |  | |  | |  | |  | |
| **Bird/GX/62/05(H9N2)** | | **H5N1-like** | |  | |  | |  | |  | |  | |  | |  | |  | |  | |  | |  | |  | |  | |  | |  | |  | |  | |  | |  | |  | |  | |  | |  | |
| **Qa/GX/B1/06(H9N2)** | | **H5N1-like** | |  | |  | |  | |  | |  | |  | |  | |  | |  | |  | |  | |  | |  | |  | |  | |  | |  | |  | |  | |  | |  | |  | |  | |
| **Bird/GX/H1/06(H9N2)** | | **H5N1-like** | | **E** | |  | |  | |  | |  | |  | |  | |  | |  | |  | | **P** | |  | |  | |  | |  | |  | |  | |  | |  | |  | |  | |  | |  | |
| **Sw/GX/S11/05(H9N2)** | | **H5N1-like** | |  | |  | |  | |  | |  | |  | |  | |  | |  | |  | |  | | **R** | |  | |  | |  | |  | |  | |  | |  | |  | |  | |  | |  | |
| **Sw/GX/S15/05(H9N2)** | | **H5N1-like** | |  | |  | |  | |  | |  | |  | |  | |  | |  | |  | |  | |  | |  | |  | |  | |  | |  | |  | |  | |  | |  | |  | |  | |
| **Ck/HK/YU22/02(H5N1)** | | **H5N1-like** | |  | |  | |  | |  | |  | |  | |  | |  | |  | |  | |  | |  | |  | |  | |  | |  | |  | |  | |  | |  | |  | |  | |  | |
| **Ck/YN/1252/03(H5N1)** | | **H5N1-like** | |  | |  | |  | |  | |  | |  | |  | |  | |  | |  | |  | |  | |  | |  | |  | |  | |  | |  | |  | |  | |  | |  | |  | |
| **Ck/HeN/01/04(H5N1)** | | **H5N1-like** | |  | |  | |  | |  | |  | |  | |  | |  | |  | |  | |  | |  | |  | |  | |  | |  | |  | |  | |  | |  | |  | |  | |  | |
| **Ck/BJ/1/94(H9N2)** | | **BJ94-like** | |  | |  | |  | |  | |  | |  | |  | |  | |  | |  | |  | |  | |  | |  | | **E** | |  | |  | | **N** | |  | |  | |  | |  | | **V** | |
| **Ck/HK/739/94(H9N2)** | | **BJ94-like** | |  | |  | |  | |  | |  | |  | |  | |  | | **I** | |  | |  | |  | |  | |  | | **E** | |  | | **D** | | **N** | |  | |  | |  | |  | |  | |
| **Ck/SD/6/96(H9N2)** | | **BJ94-like** | |  | |  | |  | |  | |  | |  | |  | |  | |  | |  | |  | |  | |  | |  | | **K** | |  | | **D** | | **N** | |  | |  | |  | |  | | **V** | |
| **Dk/NJ/2/97(H9N2)** | | **BJ94-like** | |  | |  | |  | |  | |  | |  | |  | |  | |  | |  | |  | |  | |  | |  | | **K** | |  | | **D** | | **N** | |  | |  | |  | |  | | **V** | |
| **Dk/HK/Y280/97(H9N2)** | | **BJ94-like** | |  | |  | |  | |  | | **V** | |  | |  | |  | |  | |  | |  | |  | |  | |  | | **K** | |  | | **D** | | **N** | |  | |  | |  | | **I** | | **V** | |
| **Ck/BJ/8/98(H9N2)** | | **BJ94-like** | |  | |  | |  | |  | |  | |  | |  | |  | |  | |  | |  | |  | |  | |  | | **K** | |  | | **D** | | **N** | |  | |  | |  | | **I** | | **V** | |
| **Ck/FJ/25/00(H9N2)** | | **BJ94-like** | |  | |  | |  | |  | |  | |  | |  | |  | |  | |  | |  | |  | |  | |  | | **K** | |  | | **D** | | **N** | |  | |  | | **S** | |  | | **V** | |
| **Ck/GD/4/00(H9N2)** | | **BJ94-like** | |  | |  | |  | |  | |  | |  | |  | |  | |  | |  | |  | |  | |  | |  | | **K** | |  | | **D** | | **N** | |  | |  | |  | | **I** | | **V** | |
| **Pg/NC/2-0461/00(H9N2)** | | **BJ94-like** | |  | |  | |  | |  | |  | |  | |  | |  | |  | |  | |  | |  | |  | |  | | **K** | |  | | **D** | | **N** | |  | |  | |  | | **I** | | **V** | |
| **Qa/NC/2-0460/00(H9N2)** | | **BJ94-like** | |  | |  | |  | |  | |  | |  | |  | |  | |  | |  | |  | |  | |  | |  | | **K** | |  | | **D** | | **N** | |  | |  | |  | | **I** | | **V** | |
| **WD/NC/2-0480/00(H9N2)** | | **BJ94-like** | |  | |  | |  | |  | |  | |  | |  | |  | |  | |  | |  | |  | |  | |  | | **K** | |  | | **D** | | **N** | |  | |  | |  | | **I** | | **V** | |
| **Ck/HeN/43/02(H9N2)** | | **BJ94-like** | |  | |  | |  | |  | |  | |  | |  | |  | |  | |  | |  | |  | |  | |  | | **K** | |  | | **D** | | **N** | |  | |  | |  | | **I** | | **V** | |
| **Ck/GD/6/97(H9N2)** | | **G1-like** | |  | |  | |  | | **A** | |  | |  | |  | |  | |  | |  | |  | |  | | **R** | |  | |  | |  | |  | |  | |  | | **L** | |  | |  | | **V** | |
| **Ck/HK/G23/97(H9N2)** | | **G1-like** | |  | |  | |  | | **A** | |  | |  | |  | |  | |  | |  | |  | |  | | **R** | |  | |  | |  | |  | |  | |  | | **L** | |  | |  | | **V** | |
| **Ck/HK/G9/97(H9N2)** | | **G1-like** | |  | |  | |  | | **A** | |  | |  | |  | |  | |  | |  | |  | |  | | **R** | |  | |  | |  | |  | |  | |  | | **L** | |  | |  | | **V** | |
| **Pg/HK/Y233/97(H9N2)** | | **G1-like** | |  | |  | |  | | **A** | |  | |  | |  | |  | |  | |  | |  | |  | | **R** | |  | |  | |  | |  | |  | |  | | **L** | |  | |  | | **V** | |
| **Qa/HK/G1/97(H9N2)** | | **G1-like** | |  | |  | |  | | **A** | |  | |  | |  | |  | |  | |  | |  | |  | | **R** | |  | |  | |  | |  | |  | |  | | **L** | |  | |  | | **V** | |
| **Pa/Chiba/1/97(H9N2)** | | **G1-like** | |  | |  | |  | | **A** | |  | |  | |  | | **C** | |  | |  | |  | |  | | **R** | |  | |  | |  | |  | |  | |  | | **L** | |  | |  | | **V** | |
| **Pa/Narita/92A/98(H9N2)** | | **G1-like** | |  | |  | |  | |  | |  | |  | |  | |  | |  | |  | |  | |  | | **R** | |  | |  | |  | |  | |  | |  | | **L** | |  | | **V** | | **V** | |
| **Ck/GX/10/99(H9N2)** | | **G1-like** | |  | |  | |  | | **A** | |  | |  | |  | |  | |  | |  | |  | |  | | **R** | |  | |  | |  | |  | |  | |  | | **L** | |  | |  | | **V** | |
| **Ck/GX/9/99(H9N2)** | | **G1-like** | |  | |  | |  | | **A** | |  | |  | |  | |  | |  | |  | |  | |  | | **R** | |  | |  | |  | |  | |  | |  | | **L** | |  | |  | | **V** | |
| **Ck/Pk/2/99(H9N2)** | | **G1-like** | |  | |  | |  | | **A** | |  | |  | |  | |  | |  | |  | |  | |  | | **R** | |  | |  | |  | |  | |  | |  | | **L** | |  | |  | | **V** | |
| **Ck/HLJ/35/00(H9N2)** | | **G1-like** | |  | |  | |  | | **A** | |  | |  | |  | |  | |  | |  | |  | |  | | **R** | |  | |  | |  | |  | |  | |  | | **L** | |  | |  | | **V** | |
| **HK/1073/99(H9N2)** | | **G1-like** | |  | |  | |  | | **A** | |  | |  | |  | |  | |  | |  | |  | |  | | **R** | |  | |  | |  | |  | |  | |  | | **L** | |  | |  | | **V** | |
| **HK/1074/99(H9N2)** | | **G1-like** | |  | |  | |  | | **A** | |  | |  | |  | |  | |  | |  | |  | |  | | **R** | |  | |  | |  | |  | |  | |  | | **L** | |  | |  | | **V** | |
| **GZ/333/99(H9N2)** | | **G1-like** | | ***** | | ***** | | ***** | | **A** | |  | |  | |  | |  | |  | |  | |  | |  | | **R** | |  | |  | | **L** | |  | |  | |  | | **L** | |  | |  | | **V** | |
| **HK/2108/03(H9N2)** | | **G1-like** | |  | |  | |  | | **A** | |  | |  | |  | |  | |  | |  | |  | |  | | **R** | |  | |  | |  | |  | |  | |  | | **L** | |  | |  | | **V** | |
| **Sw/GX/FS2/05(H9N2)** | | **G1-like** | | **G** | |  | |  | | **A** | |  | |  | |  | |  | |  | |  | |  | |  | | **R** | |  | |  | |  | |  | |  | |  | | **L** | |  | |  | | **V** | |
| **Ck/SH/F/98(H9N2)** | | **SH/F/98-like** | |  | |  | |  | | **A** | |  | |  | |  | |  | |  | |  | |  | |  | |  | |  | |  | |  | |  | |  | |  | |  | |  | |  | |  | |
| **Ck/HLJ/48/01(H9N2)** | | **SH/F/98-like** | |  | |  | |  | | **A** | |  | |  | | **E** | |  | |  | |  | |  | |  | |  | | **M** | |  | |  | |  | |  | |  | |  | |  | |  | |  | |
| **Ck/SH/10/01(H9N2)** | | **SH/F/98-like** | |  | |  | |  | | **A** | |  | |  | |  | |  | |  | |  | |  | |  | |  | |  | |  | |  | |  | |  | |  | |  | |  | |  | |  | |
| **Gs/GD/1/96(H5N1)** | | **SH/F/98-like** | |  | |  | |  | | **A** | |  | |  | |  | |  | |  | |  | |  | |  | |  | |  | |  | |  | |  | |  | |  | |  | |  | |  | | **V** | |
| **Dk/SH/35/02(H5N1)** | | **SH/F/98-like** | |  | |  | |  | | **A** | |  | |  | |  | |  | |  | |  | |  | |  | |  | |  | |  | |  | |  | |  | |  | |  | |  | |  | |  | |
| **Dk/HK/Y439/97(H9N2)** | | **Y439-like** | |  | |  | |  | | **A** | |  | |  | |  | |  | |  | | **X** | |  | |  | |  | |  | |  | |  | |  | |  | | **Q** | |  | |  | |  | | **V** | |
| **Ck/KR/006/96(H9N2)** | | **KR323-like** | |  | |  | |  | | **A** | |  | |  | |  | |  | |  | |  | |  | |  | |  | |  | |  | |  | |  | |  | |  | |  | |  | |  | | **V** | |
| **Ck/KR/323/96(H9N2)** | | **KR323-like** | |  | |  | | **I** | | **A** | |  | |  | |  | |  | |  | |  | |  | |  | | **R** | |  | |  | |  | |  | |  | |  | |  | |  | |  | | **V** | |
| **Ck/KR/99029/99(H9N2)** | | **KR323-like** | | ***** | | ***** | |  | | **A** | |  | | **L** | |  | |  | |  | |  | |  | |  | |  | |  | |  | |  | |  | |  | |  | |  | |  | |  | | **V** | |
| **KR/0028/00(H9N2)** | | **KR323-like** | |  | | **I** | | **I** | | **A** | |  | |  | |  | |  | |  | |  | |  | |  | |  | |  | |  | |  | |  | |  | |  | |  | |  | |  | | **V** | |
| **Dk/DE/113/95(H9N2)** | | **DE113-like** | | ***** | | ***** | | ***** | | ***** | | ***** | | ***** | | ***** | | ***** | | ***** | | ***** | | ***** | | ***** | | ***** | | ***** | |  | |  | |  | |  | |  | |  | |  | |  | | **V** | |
| **Ph/IE/PV18/97(H9N2)** | | **DE113-like** | | ***** | | ***** | | ***** | | ***** | | ***** | | ***** | | ***** | | ***** | | ***** | | ***** | | ***** | | ***** | | ***** | | ***** | | ***** | | ***** | |  | |  | |  | |  | |  | |  | | **V** | |
| **DK/NC/8-174/00(H3N6)** | | **DE113-like** | |  | |  | |  | | **A** | |  | |  | |  | |  | |  | |  | |  | |  | |  | |  | |  | |  | |  | |  | |  | |  | |  | |  | | **V** | |
| **Ty/CA/189/66(H9N2)** | | **WI/1/66-like** | |  | |  | |  | | **A** | |  | |  | |  | |  | |  | |  | |  | |  | |  | |  | |  | |  | |  | |  | |  | |  | |  | |  | | **V** | |
| **Ty/WI/1/66(H9N2)** | | **WI/1/66-like** | |  | |  | |  | | **A** | |  | |  | |  | |  | |  | |  | |  | |  | |  | |  | |  | |  | |  | |  | |  | |  | |  | |  | | **V** | |
| **virus** | **lineage** | | **Amino acid residues at different positions in the encoding regions of the PB1 protein** | | | | | | | | | | | | | | | | | | | | | | | | | | | | | | | | | | | | | | | | | | | | | |  |
|  |  |  | **121** | | **131** | | **134** | | **142** | | **143** | | **149** | | **152** | | **154** | | **156** | | **157** | | **159** | | **161** | | **171** | | **172** | | **174** | | **175** | | **177** | | **179** | | **182** | | **183** | | **191** | | **197** | | **198** | |  |
| **BbM/GX/29/05(H9N2)** | | **H5N1-like** | | **K** | | **W** | | **N** | | **A** | | **L** | | **I** | | **S** | | **G** | | **T** | | **A** | | **E** | | **G** | | **M** | | **E** | | **M** | | **D** | | **E** | | **M** | | **T** | | **T** | | **V** | | **K** | | **k** | |
| **BbM/GX/30/05(H9N2)** | | **H5N1-like** | |  | |  | |  | |  | |  | |  | |  | |  | |  | |  | |  | |  | |  | |  | |  | |  | |  | |  | |  | |  | |  | |  | |  | |
| **BbM/GX/31/05(H9N2)** | | **H5N1-like** | |  | |  | |  | |  | |  | |  | |  | |  | |  | |  | |  | |  | |  | |  | |  | |  | |  | |  | |  | |  | |  | |  | |  | |
| **Bird/GX/62/05(H9N2)** | | **H5N1-like** | |  | | **R** | |  | |  | |  | |  | |  | |  | |  | |  | |  | |  | |  | |  | |  | |  | |  | |  | |  | |  | |  | |  | |  | |
| **Qa/GX/B1/06(H9N2)** | | **H5N1-like** | |  | |  | |  | |  | |  | |  | |  | |  | |  | |  | |  | |  | |  | |  | |  | |  | |  | |  | |  | |  | |  | |  | |  | |
| **Bird/GX/H1/06(H9N2)** | | **H5N1-like** | |  | |  | |  | |  | |  | |  | |  | |  | |  | |  | |  | |  | |  | |  | |  | |  | |  | |  | |  | |  | |  | |  | |  | |
| **Sw/GX/S11/05(H9N2)** | | **H5N1-like** | |  | |  | |  | |  | |  | |  | |  | |  | |  | |  | |  | |  | |  | |  | |  | |  | |  | |  | |  | |  | |  | |  | |  | |
| **Sw/GX/S15/05(H9N2)** | | **H5N1-like** | |  | |  | |  | |  | |  | |  | |  | |  | |  | |  | |  | |  | |  | |  | |  | |  | |  | |  | |  | |  | |  | |  | |  | |
| **Ck/HK/YU22/02(H5N1)** | | **H5N1-like** | |  | |  | |  | |  | |  | |  | |  | |  | |  | |  | |  | |  | |  | |  | |  | |  | |  | |  | |  | |  | |  | |  | |  | |
| **Ck/YN/1252/03(H5N1)** | | **H5N1-like** | |  | |  | |  | |  | |  | |  | |  | |  | |  | |  | |  | |  | |  | |  | |  | |  | |  | |  | |  | |  | |  | |  | |  | |
| **Ck/HeN/01/04(H5N1)** | | **H5N1-like** | |  | |  | |  | |  | |  | |  | |  | |  | |  | |  | |  | |  | |  | |  | |  | |  | |  | |  | |  | |  | |  | |  | |  | |
| **Ck/BJ/1/94(H9N2)** | | **BJ94-like** | |  | |  | |  | |  | |  | | **V** | |  | |  | |  | | **T** | |  | |  | | **T** | | **D** | |  | | **N** | |  | |  | |  | |  | |  | |  | |  | |
| **Ck/HK/739/94(H9N2)** | | **BJ94-like** | |  | |  | |  | |  | |  | | **V** | |  | |  | |  | | **T** | |  | |  | | **T** | | **D** | |  | | **N** | |  | |  | |  | |  | |  | |  | |  | |
| **Ck/SD/6/96(H9N2)** | | **BJ94-like** | |  | |  | |  | |  | | **F** | | **V** | |  | |  | |  | | **I** | |  | |  | | **T** | | **D** | |  | | **N** | |  | |  | |  | |  | |  | |  | |  | |
| **Dk/NJ/2/97(H9N2)** | | **BJ94-like** | |  | |  | |  | |  | |  | | **V** | |  | |  | |  | | **I** | |  | |  | | **T** | | **D** | |  | | **N** | |  | |  | |  | |  | |  | |  | |  | |
| **Dk/HK/Y280/97(H9N2)** | | **BJ94-like** | |  | |  | |  | |  | |  | | **V** | |  | |  | |  | | **T** | |  | | **R** | |  | | **D** | |  | | **N** | |  | |  | |  | |  | |  | |  | |  | |
| **Ck/BJ/8/98(H9N2)** | | **BJ94-like** | |  | |  | |  | |  | |  | | **V** | |  | |  | |  | | **T** | |  | |  | | **T** | | **D** | |  | | **N** | |  | |  | |  | | **I** | |  | |  | |  | |
| **Ck/FJ/25/00(H9N2)** | | **BJ94-like** | |  | |  | |  | |  | |  | | **V** | |  | |  | |  | | **T** | |  | |  | | **A** | | **D** | |  | | **N** | |  | |  | |  | |  | |  | |  | |  | |
| **Ck/GD/4/00(H9N2)** | | **BJ94-like** | |  | |  | |  | |  | |  | | **V** | |  | |  | |  | | **T** | |  | |  | |  | | **D** | |  | | **N** | |  | |  | |  | |  | |  | |  | | **N** | |
| **Pg/NC/2-0461/00(H9N2)** | | **BJ94-like** | |  | |  | |  | |  | |  | | **V** | |  | |  | |  | | **T** | |  | |  | |  | | **D** | |  | | **N** | |  | |  | |  | |  | |  | |  | |  | |
| **Qa/NC/2-0460/00(H9N2)** | | **BJ94-like** | |  | |  | |  | |  | |  | | **V** | |  | |  | |  | | **T** | |  | |  | |  | | **D** | |  | | **N** | |  | |  | |  | |  | | **I** | |  | |  | |
| **WD/NC/2-0480/00(H9N2)** | | **BJ94-like** | |  | |  | |  | |  | |  | | **V** | |  | |  | |  | | **T** | |  | |  | |  | | **D** | |  | | **N** | |  | |  | |  | |  | |  | |  | |  | |
| **Ck/HeN/43/02(H9N2)** | | **BJ94-like** | |  | |  | |  | |  | |  | | **V** | |  | |  | |  | | **T** | |  | |  | |  | | **D** | |  | | **N** | |  | |  | |  | |  | |  | | **R** | |  | |
| **Ck/GD/6/97(H9N2)** | | **G1-like** | |  | |  | |  | |  | |  | | **V** | |  | |  | |  | |  | |  | |  | |  | |  | |  | |  | |  | |  | |  | |  | |  | |  | |  | |
| **Ck/HK/G23/97(H9N2)** | | **G1-like** | |  | |  | |  | |  | |  | | **V** | |  | |  | |  | |  | |  | |  | |  | |  | |  | |  | |  | |  | |  | |  | |  | |  | |  | |
| **Ck/HK/G9/97(H9N2)** | | **G1-like** | |  | |  | |  | |  | |  | | **V** | |  | |  | |  | |  | |  | |  | |  | |  | |  | |  | |  | |  | |  | |  | |  | |  | |  | |
| **Pg/HK/Y233/97(H9N2)** | | **G1-like** | |  | |  | |  | | **S** | |  | | **V** | |  | |  | |  | |  | |  | |  | |  | |  | |  | |  | |  | |  | |  | |  | |  | |  | |  | |
| **Qa/HK/G1/97(H9N2)** | | **G1-like** | |  | |  | |  | |  | |  | | **V** | |  | |  | |  | |  | |  | |  | |  | |  | |  | |  | |  | |  | |  | |  | |  | |  | |  | |
| **Pa/Chiba/1/97(H9N2)** | | **G1-like** | |  | |  | |  | |  | |  | | **V** | |  | |  | |  | |  | |  | |  | |  | |  | |  | |  | |  | |  | |  | |  | |  | |  | |  | |
| **Pa/Narita/92A/98(H9N2)** | | **G1-like** | |  | |  | |  | |  | |  | | **V** | |  | |  | |  | |  | |  | |  | |  | |  | |  | |  | |  | |  | |  | |  | |  | |  | |  | |
| **Ck/GX/10/99(H9N2)** | | **G1-like** | |  | |  | |  | |  | |  | | **V** | |  | | **S** | |  | |  | |  | |  | |  | |  | |  | |  | |  | |  | |  | |  | |  | |  | |  | |
| **Ck/GX/9/99(H9N2)** | | **G1-like** | |  | |  | |  | |  | |  | | **V** | |  | | **S** | |  | |  | |  | |  | |  | |  | |  | |  | |  | |  | |  | |  | |  | |  | |  | |
| **Ck/Pk/2/99(H9N2)** | | **G1-like** | |  | |  | |  | |  | |  | | **V** | |  | |  | |  | |  | |  | |  | |  | |  | |  | |  | |  | |  | |  | |  | |  | |  | |  | |
| **Ck/HLJ/35/00(H9N2)** | | **G1-like** | | **R** | |  | |  | |  | |  | | **V** | |  | | **S** | |  | |  | | **D** | |  | |  | |  | |  | |  | |  | |  | |  | |  | |  | |  | |  | |
| **HK/1073/99(H9N2)** | | **G1-like** | |  | |  | |  | |  | |  | | **V** | |  | |  | |  | |  | |  | |  | |  | |  | |  | |  | |  | |  | |  | |  | |  | |  | |  | |
| **HK/1074/99(H9N2)** | | **G1-like** | |  | |  | |  | |  | |  | | **V** | |  | |  | |  | |  | |  | |  | |  | |  | |  | |  | |  | |  | |  | |  | |  | |  | |  | |
| **GZ/333/99(H9N2)** | | **G1-like** | |  | |  | |  | |  | |  | | **V** | |  | | **S** | |  | |  | |  | |  | |  | |  | |  | |  | |  | |  | |  | |  | |  | |  | |  | |
| **HK/2108/03(H9N2)** | | **G1-like** | |  | |  | |  | |  | |  | | **V** | |  | | **S** | |  | |  | |  | |  | |  | |  | |  | |  | |  | |  | |  | |  | |  | |  | |  | |
| **Sw/GX/FS2/05(H9N2)** | | **G1-like** | |  | |  | |  | |  | |  | | **V** | |  | |  | |  | |  | | **K** | |  | |  | |  | |  | |  | |  | |  | |  | |  | |  | |  | |  | |
| **Ck/SH/F/98(H9N2)** | | **SH/F/98-like** | |  | |  | |  | |  | |  | | **V** | |  | |  | |  | |  | |  | |  | |  | |  | |  | |  | |  | |  | |  | |  | |  | |  | |  | |
| **Ck/HLJ/48/01(H9N2)** | | **SH/F/98-like** | |  | |  | |  | |  | |  | | **V** | |  | |  | |  | |  | |  | |  | |  | |  | |  | |  | |  | | **I** | |  | |  | |  | |  | |  | |
| **Ck/SH/10/01(H9N2)** | | **SH/F/98-like** | |  | |  | |  | |  | |  | | **V** | |  | |  | |  | |  | |  | |  | |  | |  | |  | |  | |  | |  | |  | |  | |  | |  | |  | |
| **Gs/GD/1/96(H5N1)** | | **SH/F/98-like** | |  | |  | | **K** | |  | |  | | **V** | |  | |  | |  | |  | |  | |  | |  | |  | |  | |  | | **G** | |  | | **I** | |  | |  | |  | |  | |
| **Dk/SH/35/02(H5N1)** | | **SH/F/98-like** | |  | |  | |  | |  | |  | | **V** | |  | |  | |  | |  | |  | |  | |  | |  | |  | |  | |  | |  | |  | |  | |  | |  | |  | |
| **Dk/HK/Y439/97(H9N2)** | | **Y439-like** | |  | |  | |  | |  | |  | | **V** | |  | |  | |  | |  | |  | |  | |  | |  | |  | |  | |  | |  | |  | |  | |  | |  | |  | |
| **Ck/KR/006/96(H9N2)** | | **KR323-like** | |  | |  | |  | |  | |  | | **V** | |  | |  | |  | |  | |  | |  | |  | |  | |  | |  | |  | |  | |  | |  | |  | |  | |  | |
| **Ck/KR/323/96(H9N2)** | | **KR323-like** | |  | |  | |  | |  | |  | | **V** | |  | |  | |  | |  | |  | |  | |  | |  | |  | |  | |  | |  | |  | |  | |  | |  | |  | |
| **Ck/KR/99029/99(H9N2)** | | **KR323-like** | |  | |  | |  | |  | |  | | **V** | |  | |  | |  | |  | |  | |  | |  | |  | |  | |  | |  | |  | |  | |  | |  | |  | |  | |
| **KR/0028/00(H9N2)** | | **KR323-like** | |  | |  | |  | |  | |  | | **V** | |  | |  | |  | |  | |  | |  | |  | | **G** | |  | |  | |  | | **V** | |  | |  | |  | |  | |  | |
| **Dk/DE/113/95(H9N2)** | | **DE113-like** | |  | |  | |  | |  | |  | | **V** | |  | |  | |  | |  | |  | |  | |  | |  | | **I** | |  | |  | |  | |  | |  | |  | |  | |  | |
| **Ph/IE/PV18/97(H9N2)** | | **DE113-like** | |  | |  | |  | |  | |  | | **V** | | **T** | |  | |  | |  | |  | |  | |  | |  | |  | |  | |  | |  | |  | |  | |  | |  | |  | |
| **DK/NC/8-174/00(H3N6)** | | **DE113-like** | |  | |  | |  | |  | |  | | **V** | |  | |  | |  | |  | |  | |  | |  | |  | |  | |  | |  | |  | |  | |  | |  | |  | |  | |
| **Ty/CA/189/66(H9N2)** | | **WI/1/66-like** | |  | |  | |  | |  | |  | | **V** | |  | |  | | **A** | |  | |  | |  | |  | |  | |  | |  | |  | |  | |  | |  | |  | |  | |  | |
| **Ty/WI/1/66(H9N2)** | | **WI/1/66-like** | |  | |  | |  | |  | |  | | **V** | |  | |  | |  | |  | |  | |  | |  | |  | |  | |  | |  | |  | |  | |  | |  | |  | |  | |

| **virus** | | | **lineage** | **Amino acid residues at different positions in the encoding regions of the PB1 protein** | | | | | | | | | | | | | | | | | | | | | | | | | | | | | | | | | | | | | | | | | | | | | |  |
| --- | --- | --- | --- | --- | --- | --- | --- | --- | --- | --- | --- | --- | --- | --- | --- | --- | --- | --- | --- | --- | --- | --- | --- | --- | --- | --- | --- | --- | --- | --- | --- | --- | --- | --- | --- | --- | --- | --- | --- | --- | --- | --- | --- | --- | --- | --- | --- | --- | --- | --- |
|  |  |  |  | **200** | | **202** | | **203** | | **207** | | **211** | | **213** | | **214** | | **215** | | **217** | | **237** | | **239** | | **253** | | **257** | | **261** | | **265** | | **274** | | **278** | | **293** | | **302** | | **306** | | **308** | | **317** | | **336** | |  |
| **BbM/GX/29/05(H9N2)** | | **H5N1-like** | | | **V** | | **Q** | | **R** | | **K** | | **R** | | **N** | | **K** | | **K** | | **Y** | | **k** | | **R** | | **Y** | | **T** | | **S** | | **k** | | **G** | | **K** | | **S** | | **I** | | **N** | | **K** | | **M** | | **V** | |
| **BbM/GX/30/05(H9N2)** | | **H5N1-like** | | |  | |  | |  | |  | |  | |  | |  | |  | |  | |  | |  | |  | |  | |  | |  | |  | |  | |  | |  | |  | |  | |  | |  | |
| **BbM/GX/31/05(H9N2)** | | **H5N1-like** | | |  | |  | |  | |  | |  | |  | |  | |  | |  | |  | |  | |  | |  | |  | |  | |  | |  | |  | |  | |  | |  | |  | |  | |
| **Bird/GX/62/05(H9N2)** | | **H5N1-like** | | |  | |  | |  | |  | |  | |  | |  | |  | |  | |  | |  | |  | |  | |  | |  | |  | |  | |  | |  | |  | |  | |  | |  | |
| **Qa/GX/B1/06(H9N2)** | | **H5N1-like** | | | **A** | |  | |  | |  | |  | |  | |  | |  | |  | |  | |  | |  | |  | |  | |  | |  | |  | |  | |  | |  | |  | |  | |  | |
| **Bird/GX/H1/06(H9N2)** | | **H5N1-like** | | |  | |  | |  | |  | |  | |  | |  | |  | |  | |  | |  | |  | |  | |  | |  | |  | |  | |  | |  | |  | |  | |  | |  | |
| **Sw/GX/S11/05(H9N2)** | | **H5N1-like** | | |  | |  | |  | |  | |  | |  | |  | |  | |  | |  | |  | |  | |  | |  | |  | |  | |  | |  | |  | |  | | **E** | |  | |  | |
| **Sw/GX/S15/05(H9N2)** | | **H5N1-like** | | |  | |  | |  | |  | |  | |  | |  | |  | |  | |  | |  | |  | |  | |  | |  | |  | |  | |  | |  | |  | |  | |  | |  | |
| **Ck/HK/YU22/02(H5N1)** | | **H5N1-like** | | |  | |  | |  | |  | |  | |  | |  | |  | |  | |  | |  | |  | |  | |  | |  | |  | |  | |  | |  | |  | |  | |  | |  | |
| **Ck/YN/1252/03(H5N1)** | | **H5N1-like** | | |  | |  | |  | |  | |  | |  | |  | |  | |  | |  | |  | |  | |  | |  | |  | |  | |  | |  | |  | |  | |  | |  | |  | |
| **Ck/HeN/01/04(H5N1)** | | **H5N1-like** | | |  | |  | |  | |  | |  | |  | |  | |  | |  | |  | |  | |  | |  | |  | |  | |  | |  | |  | |  | |  | |  | |  | |  | |
| **Ck/BJ/1/94(H9N2)** | | **BJ94-like** | | |  | |  | |  | |  | |  | |  | | **R** | | **R** | |  | |  | |  | |  | |  | |  | |  | |  | |  | |  | |  | |  | |  | | **V** | |  | |
| **Ck/HK/739/94(H9N2)** | | **BJ94-like** | | |  | | **H** | |  | |  | |  | |  | | **R** | | **R** | |  | | **E** | |  | |  | |  | |  | |  | |  | |  | |  | |  | |  | |  | | **V** | |  | |
| **Ck/SD/6/96(H9N2)** | | **BJ94-like** | | |  | |  | |  | |  | |  | |  | | **R** | | **R** | |  | |  | |  | |  | |  | |  | |  | |  | |  | |  | |  | |  | |  | | **V** | |  | |
| **Dk/NJ/2/97(H9N2)** | | **BJ94-like** | | |  | |  | |  | |  | |  | |  | | **R** | | **R** | |  | |  | |  | |  | |  | |  | |  | |  | |  | |  | |  | |  | |  | | **V** | |  | |
| **Dk/HK/Y280/97(H9N2)** | | **BJ94-like** | | |  | | **H** | |  | |  | |  | |  | | **R** | | **R** | |  | |  | |  | |  | |  | |  | |  | |  | |  | |  | |  | |  | |  | | **V** | |  | |
| **Ck/BJ/8/98(H9N2)** | | **BJ94-like** | | |  | |  | |  | |  | |  | |  | | **R** | | **R** | |  | |  | |  | |  | |  | |  | |  | |  | |  | |  | |  | |  | |  | | **V** | |  | |
| **Ck/FJ/25/00(H9N2)** | | **BJ94-like** | | |  | |  | |  | |  | |  | |  | | **R** | | **R** | |  | |  | |  | |  | |  | |  | |  | |  | |  | |  | |  | |  | |  | | **V** | | **I** | |
| **Ck/GD/4/00(H9N2)** | | **BJ94-like** | | |  | |  | | **S** | |  | |  | |  | | **R** | | **R** | |  | |  | |  | |  | |  | |  | |  | |  | |  | |  | |  | |  | |  | | **V** | |  | |
| **Pg/NC/2-0461/00(H9N2)** | | **BJ94-like** | | |  | |  | |  | |  | |  | |  | | **R** | | **R** | |  | |  | |  | |  | |  | |  | |  | |  | |  | |  | |  | |  | |  | | **V** | |  | |
| **Qa/NC/2-0460/00(H9N2)** | | **BJ94-like** | | |  | |  | |  | |  | |  | |  | | **R** | | **R** | |  | | **R** | |  | |  | |  | |  | |  | |  | |  | |  | |  | |  | |  | | **V** | |  | |
| **WD/NC/2-0480/00(H9N2)** | | **BJ94-like** | | |  | |  | |  | |  | |  | |  | | **R** | | **R** | |  | |  | |  | |  | |  | |  | |  | |  | |  | |  | |  | |  | |  | | **V** | |  | |
| **Ck/HeN/43/02(H9N2)** | | **BJ94-like** | | |  | |  | |  | |  | |  | |  | |  | | **R** | | **H** | |  | |  | |  | | **A** | |  | |  | |  | |  | |  | |  | |  | |  | | **V** | |  | |
| **Ck/GD/6/97(H9N2)** | | **G1-like** | | |  | |  | |  | |  | |  | | **T** | |  | |  | |  | |  | |  | | **H** | | **A** | |  | |  | |  | |  | |  | | **V** | |  | |  | | **I** | |  | |
| **Ck/HK/G23/97(H9N2)** | | **G1-like** | | |  | |  | |  | |  | |  | | **T** | |  | |  | |  | |  | |  | | **H** | | **A** | |  | |  | |  | |  | |  | | **V** | |  | |  | | **I** | |  | |
| **Ck/HK/G9/97(H9N2)** | | **G1-like** | | |  | |  | |  | |  | |  | | **T** | |  | |  | |  | |  | |  | | **H** | | **A** | |  | |  | |  | |  | |  | | **V** | |  | |  | | **I** | |  | |
| **Pg/HK/Y233/97(H9N2)** | | **G1-like** | | |  | |  | |  | |  | |  | | **T** | |  | |  | |  | |  | |  | | **H** | | **A** | |  | |  | |  | |  | |  | | **V** | |  | |  | | **I** | |  | |
| **Qa/HK/G1/97(H9N2)** | | **G1-like** | | |  | |  | |  | |  | | **K** | | **T** | |  | |  | |  | |  | |  | | **H** | | **A** | |  | |  | |  | |  | |  | | **V** | |  | |  | | **I** | |  | |
| **Pa/Chiba/1/97(H9N2)** | | **G1-like** | | |  | |  | |  | |  | | **K** | | **T** | |  | |  | |  | |  | |  | | **H** | | **A** | |  | |  | |  | |  | |  | | **V** | |  | |  | | **I** | |  | |
| **Pa/Narita/92A/98(H9N2)** | | **G1-like** | | |  | |  | |  | |  | | **K** | | **T** | |  | |  | |  | |  | |  | | **H** | | **A** | |  | |  | |  | |  | |  | | **V** | |  | |  | | **I** | |  | |
| **Ck/GX/10/99(H9N2)** | | **G1-like** | | |  | |  | |  | |  | | **K** | | **T** | |  | |  | |  | |  | |  | | **H** | | **A** | |  | |  | |  | |  | |  | | **V** | |  | |  | | **I** | |  | |
| **Ck/GX/9/99(H9N2)** | | **G1-like** | | |  | |  | |  | |  | | **K** | | **T** | |  | |  | |  | |  | |  | | **H** | | **A** | |  | |  | |  | |  | |  | | **V** | |  | |  | | **I** | |  | |
| **Ck/Pk/2/99(H9N2)** | | **G1-like** | | |  | |  | |  | |  | | **K** | | **T** | |  | |  | |  | |  | |  | | **H** | | **A** | |  | |  | |  | |  | |  | | **V** | |  | |  | | **I** | |  | |
| **Ck/HLJ/35/00(H9N2)** | | **G1-like** | | |  | |  | |  | |  | | **K** | | **T** | |  | |  | |  | |  | |  | | **H** | | **A** | |  | |  | |  | |  | |  | | **V** | |  | |  | | **I** | |  | |
| **HK/1073/99(H9N2)** | | **G1-like** | | |  | |  | |  | |  | | **K** | | **T** | |  | |  | |  | |  | |  | | **H** | | **A** | |  | |  | |  | |  | |  | | **V** | |  | |  | | **I** | |  | |
| **HK/1074/99(H9N2)** | | **G1-like** | | |  | |  | |  | |  | |  | | **T** | |  | |  | |  | |  | |  | | **H** | | **A** | |  | |  | |  | |  | |  | | **V** | |  | |  | | **I** | |  | |
| **GZ/333/99(H9N2)** | | **G1-like** | | |  | |  | |  | |  | | **K** | | **T** | |  | |  | |  | |  | |  | | **H** | | **A** | |  | |  | |  | | **N** | |  | | **V** | |  | |  | | **I** | |  | |
| **HK/2108/03(H9N2)** | | **G1-like** | | |  | |  | |  | |  | | **K** | | **T** | |  | |  | |  | |  | |  | | **H** | | **A** | |  | |  | |  | |  | |  | | **V** | |  | |  | | **I** | |  | |
| **Sw/GX/FS2/05(H9N2)** | | **G1-like** | | |  | |  | |  | |  | | **K** | | **T** | |  | |  | |  | |  | | **G** | | **H** | | **A** | |  | |  | |  | |  | |  | | **V** | |  | |  | | **I** | |  | |
| **Ck/SH/F/98(H9N2)** | | **SH/F/98-like** | | |  | |  | |  | |  | |  | |  | |  | | **R** | |  | |  | |  | |  | |  | |  | |  | |  | |  | |  | |  | |  | |  | |  | |  | |
| **Ck/HLJ/48/01(H9N2)** | | **SH/F/98-like** | | |  | |  | |  | |  | |  | | **T** | |  | | **R** | |  | |  | |  | |  | |  | |  | |  | |  | |  | |  | |  | |  | |  | |  | |  | |
| **Ck/SH/10/01(H9N2)** | | **SH/F/98-like** | | |  | |  | |  | |  | |  | |  | |  | | **R** | |  | |  | |  | |  | |  | |  | |  | |  | |  | |  | |  | |  | |  | |  | |  | |
| **Gs/GD/1/96(H5N1)** | | **SH/F/98-like** | | |  | |  | |  | |  | |  | |  | |  | | **R** | |  | |  | |  | |  | |  | |  | |  | |  | |  | |  | |  | |  | |  | |  | |  | |
| **Dk/SH/35/02(H5N1)** | | **SH/F/98-like** | | |  | |  | |  | |  | |  | |  | |  | | **R** | |  | |  | |  | |  | |  | |  | |  | |  | |  | |  | |  | | **S** | |  | |  | |  | |
| **Dk/HK/Y439/97(H9N2)** | | **Y439-like** | | |  | |  | |  | |  | |  | |  | |  | | **R** | |  | |  | |  | |  | |  | |  | |  | |  | |  | | **L** | |  | |  | |  | |  | |  | |
| **Ck/KR/006/96(H9N2)** | | **KR323-like** | | |  | |  | |  | |  | |  | |  | | **R** | | **R** | |  | |  | |  | |  | |  | | **N** | |  | |  | |  | |  | |  | |  | |  | | **V** | |  | |
| **Ck/KR/323/96(H9N2)** | | **KR323-like** | | |  | |  | |  | |  | |  | |  | |  | | **R** | |  | |  | |  | |  | | **H** | |  | | **E** | | **R** | |  | |  | |  | |  | |  | |  | |  | |
| **Ck/KR/99029/99(H9N2)** | | **KR323-like** | | |  | |  | |  | |  | |  | |  | |  | | **R** | |  | |  | |  | |  | |  | |  | |  | |  | |  | |  | |  | |  | |  | |  | |  | |
| **KR/0028/00(H9N2)** | | **KR323-like** | | |  | |  | |  | |  | |  | |  | |  | | **R** | |  | |  | |  | |  | | **H** | |  | |  | |  | |  | |  | |  | |  | |  | |  | |  | |
| **Dk/DE/113/95(H9N2)** | | **DE113-like** | | |  | |  | |  | |  | |  | |  | |  | | **R** | |  | |  | |  | |  | |  | |  | |  | |  | |  | |  | |  | |  | |  | |  | |  | |
| **Ph/IE/PV18/97(H9N2)** | | **DE113-like** | | |  | |  | |  | |  | |  | |  | |  | | **R** | |  | |  | |  | |  | |  | |  | |  | |  | |  | |  | |  | |  | |  | |  | |  | |
| **DK/NC/8-174/00(H3N6)** | | **DE113-like** | | |  | |  | |  | |  | |  | |  | |  | | **R** | |  | |  | |  | |  | |  | |  | |  | |  | |  | |  | |  | |  | |  | |  | |  | |
| **Ty/CA/189/66(H9N2)** | | **WI/1/66-like** | | |  | |  | |  | | **Q** | |  | |  | |  | | **R** | |  | |  | |  | |  | |  | |  | |  | |  | |  | |  | |  | |  | |  | |  | |  | |
| **Ty/WI/1/66(H9N2)** | | **WI/1/66-like** | | |  | |  | |  | |  | |  | |  | |  | | **R** | |  | |  | |  | |  | |  | |  | |  | |  | |  | |  | |  | |  | |  | |  | |  | |
| **virus** | | | **lineage** | **Amino acid residues at different positions in the encoding regions of the PB1 protein** | | | | | | | | | | | | | | | | | | | | | | | | | | | | | | | | | | | | | | | | | | | | | |  |
|  |  |  |  | **344** | | **348** | | **349** | | **350** | | **353** | | **360** | | **369** | | **370** | | **375** | | **378** | | **380** | | **383** | | **384** | | **385** | | **386** | | **387** | | **388** | | **390** | | **391** | | **397** | | **398** | | **402** | | **430** | |  |
| **BbM/GX/29/05(H9N2)** | **H5N1-like** | | | **F** | | **M** | | **A** | | **R** | | **K** | | **K** | | **P** | | **A** | | **N** | | **L** | | **Y** | | **E** | | **L** | | **T** | | **K** | | **R** | | **K** | | **E** | | **k** | | **I** | | **D** | | **S** | | **R** | |  |
| **BbM/GX/30/05(H9N2)** | **H5N1-like** | | |  | |  | |  | |  | |  | |  | |  | |  | |  | |  | |  | |  | |  | |  | |  | |  | |  | |  | |  | |  | |  | |  | |  | |  |
| **BbM/GX/31/05(H9N2)** | **H5N1-like** | | |  | |  | |  | |  | |  | |  | |  | |  | |  | |  | |  | |  | |  | |  | |  | |  | |  | |  | |  | |  | |  | |  | |  | |  |
| **Bird/GX/62/05(H9N2)** | **H5N1-like** | | |  | |  | |  | |  | |  | |  | |  | |  | |  | |  | |  | |  | | **S** | |  | | **R** | |  | |  | |  | |  | |  | |  | |  | | **K** | |  |
| **Qa/GX/B1/06(H9N2)** | **H5N1-like** | | |  | |  | |  | |  | |  | |  | |  | |  | |  | |  | |  | |  | |  | |  | |  | |  | |  | |  | |  | |  | |  | |  | |  | |  |
| **Bird/GX/H1/06(H9N2)** | **H5N1-like** | | |  | |  | |  | |  | |  | |  | |  | |  | |  | |  | |  | |  | |  | |  | |  | |  | |  | |  | |  | |  | |  | |  | |  | |  |
| **Sw/GX/S11/05(H9N2)** | **H5N1-like** | | |  | |  | |  | |  | |  | |  | |  | |  | |  | |  | |  | |  | |  | |  | |  | |  | |  | |  | |  | |  | |  | |  | |  | |  |
| **Sw/GX/S15/05(H9N2)** | **H5N1-like** | | |  | |  | |  | |  | |  | |  | |  | |  | |  | |  | |  | |  | |  | |  | |  | |  | |  | |  | |  | |  | |  | |  | |  | |  |
| **Ck/HK/YU22/02(H5N1)** | **H5N1-like** | | |  | |  | |  | |  | |  | |  | |  | |  | |  | |  | |  | |  | |  | |  | |  | | **K** | |  | | **D** | |  | |  | |  | |  | |  | |  |
| **Ck/YN/1252/03(H5N1)** | **H5N1-like** | | |  | |  | |  | |  | |  | |  | |  | |  | |  | |  | |  | |  | |  | |  | |  | | **K** | |  | |  | |  | |  | |  | |  | |  | |  |
| **Ck/HeN/01/04(H5N1)** | **H5N1-like** | | |  | |  | |  | |  | |  | |  | |  | |  | |  | |  | |  | |  | |  | |  | |  | | **K** | |  | |  | |  | |  | |  | |  | |  | |  |
| **Ck/BJ/1/94(H9N2)** | **BJ94-like** | | |  | |  | |  | |  | |  | |  | |  | |  | | **T** | |  | |  | |  | | **S** | |  | | **R** | | **K** | |  | |  | |  | |  | | **E** | |  | |  | |  |
| **Ck/HK/739/94(H9N2)** | **BJ94-like** | | |  | |  | |  | |  | |  | |  | |  | |  | | **T** | |  | |  | |  | | **S** | |  | | **R** | | **K** | |  | |  | | **R** | |  | | **E** | |  | |  | |  |
| **Ck/SD/6/96(H9N2)** | **BJ94-like** | | |  | |  | |  | |  | |  | |  | |  | |  | | **T** | |  | |  | |  | | **S** | |  | | **R** | | **K** | |  | |  | |  | |  | | **E** | |  | |  | |  |
| **Dk/NJ/2/97(H9N2)** | **BJ94-like** | | |  | |  | |  | |  | |  | |  | |  | |  | | **T** | |  | |  | |  | | **S** | |  | | **R** | | **K** | |  | |  | |  | |  | | **E** | |  | |  | |  |
| **Dk/HK/Y280/97(H9N2)** | **BJ94-like** | | |  | |  | |  | |  | |  | |  | |  | |  | | **T** | |  | |  | |  | | **S** | |  | | **R** | | **Q** | |  | |  | |  | |  | | **E** | |  | |  | |  |
| **Ck/BJ/8/98(H9N2)** | **BJ94-like** | | |  | |  | |  | |  | |  | |  | |  | |  | | **T** | |  | |  | |  | | **S** | |  | | **R** | | **Q** | |  | |  | |  | |  | | **E** | |  | |  | |  |
| **Ck/FJ/25/00(H9N2)** | **BJ94-like** | | |  | | **V** | |  | |  | |  | |  | |  | |  | | **T** | |  | |  | |  | | **S** | |  | | **R** | | **K** | |  | |  | |  | | **L** | | **E** | |  | |  | |  |
| **Ck/GD/4/00(H9N2)** | **BJ94-like** | | |  | |  | |  | |  | |  | | **R** | |  | |  | | **T** | |  | |  | |  | | **S** | |  | | **R** | | **Q** | |  | |  | |  | |  | | **E** | |  | |  | |  |
| **Pg/NC/2-0461/00(H9N2)** | **BJ94-like** | | |  | |  | |  | |  | |  | |  | |  | |  | | **T** | |  | |  | |  | | **S** | |  | | **R** | | **Q** | |  | |  | |  | |  | | **E** | |  | |  | |  |
| **Qa/NC/2-0460/00(H9N2)** | **BJ94-like** | | |  | |  | |  | |  | |  | |  | |  | |  | | **T** | |  | |  | | **G** | | **S** | |  | | **R** | | **Q** | |  | |  | |  | |  | | **E** | |  | |  | |  |
| **WD/NC/2-0480/00(H9N2)** | **BJ94-like** | | |  | |  | |  | |  | |  | |  | |  | |  | | **T** | |  | |  | |  | | **S** | |  | | **R** | | **Q** | |  | |  | |  | |  | | **E** | |  | |  | |  |
| **Ck/HeN/43/02(H9N2)** | **BJ94-like** | | |  | |  | |  | |  | |  | |  | |  | |  | | **T** | |  | |  | |  | | **S** | |  | | **R** | | **Q** | |  | |  | |  | |  | | **E** | |  | |  | |  |
| **Ck/GD/6/97(H9N2)** | **G1-like** | | |  | |  | |  | |  | |  | |  | |  | |  | |  | |  | |  | |  | | **S** | |  | | **R** | | **K** | |  | |  | |  | | **T** | | **E** | |  | |  | |  |
| **Ck/HK/G23/97(H9N2)** | **G1-like** | | |  | |  | |  | |  | |  | |  | |  | |  | |  | |  | |  | |  | | **S** | |  | | **R** | | **K** | |  | |  | |  | | **T** | | **E** | |  | |  | |  |
| **Ck/HK/G9/97(H9N2)** | **G1-like** | | | **V** | |  | |  | |  | |  | |  | |  | |  | |  | |  | |  | |  | | **S** | |  | | **R** | | **K** | |  | |  | |  | | **T** | | **E** | |  | |  | |  |
| **Pg/HK/Y233/97(H9N2)** | **G1-like** | | |  | |  | |  | |  | |  | |  | |  | |  | |  | |  | | **C** | |  | | **S** | | **S** | | **R** | | **K** | |  | |  | |  | | **T** | | **E** | |  | |  | |  |
| **Qa/HK/G1/97(H9N2)** | **G1-like** | | |  | |  | |  | |  | |  | |  | |  | |  | |  | |  | |  | |  | | **S** | |  | | **R** | | **K** | |  | |  | |  | |  | | **E** | |  | |  | |  |
| **Pa/Chiba/1/97(H9N2)** | **G1-like** | | |  | |  | |  | |  | |  | |  | | **Q** | |  | |  | |  | |  | |  | | **S** | |  | | **R** | | **K** | |  | |  | |  | |  | | **E** | |  | |  | |  |
| **Pa/Narita/92A/98(H9N2)** | **G1-like** | | |  | |  | |  | |  | |  | |  | | **Q** | |  | |  | |  | |  | |  | | **S** | |  | | **R** | | **K** | |  | |  | |  | |  | | **E** | |  | |  | |  |
| **Ck/GX/10/99(H9N2)** | **G1-like** | | |  | |  | |  | |  | |  | |  | |  | |  | |  | |  | |  | |  | | **S** | |  | | **R** | | **K** | |  | |  | |  | |  | | **E** | |  | |  | |  |
| **Ck/GX/9/99(H9N2)** | **G1-like** | | |  | |  | |  | |  | |  | |  | |  | |  | |  | |  | |  | |  | | **S** | |  | | **R** | | **K** | |  | |  | |  | |  | | **E** | |  | |  | |  |
| **Ck/Pk/2/99(H9N2)** | **G1-like** | | |  | |  | |  | |  | |  | |  | | **Q** | |  | |  | |  | |  | |  | | **S** | |  | | **R** | | **K** | |  | |  | |  | |  | | **E** | |  | |  | |  |
| **Ck/HLJ/35/00(H9N2)** | **G1-like** | | |  | |  | |  | |  | |  | |  | |  | |  | |  | |  | |  | |  | | **S** | |  | | **R** | | **K** | |  | |  | |  | |  | | **E** | |  | |  | |  |
| **HK/1073/99(H9N2)** | **G1-like** | | |  | |  | |  | |  | |  | |  | |  | |  | |  | |  | |  | |  | | **S** | |  | | **R** | | **K** | |  | |  | |  | |  | | **E** | |  | |  | |  |
| **HK/1074/99(H9N2)** | **G1-like** | | |  | |  | |  | |  | |  | | **R** | |  | |  | |  | |  | |  | |  | | **S** | |  | | **R** | | **K** | |  | |  | |  | |  | | **E** | |  | |  | |  |
| **GZ/333/99(H9N2)** | **G1-like** | | |  | |  | |  | |  | |  | |  | |  | |  | |  | |  | |  | |  | | **S** | |  | | **R** | | **K** | |  | |  | |  | |  | | **E** | |  | |  | |  |
| **HK/2108/03(H9N2)** | **G1-like** | | |  | |  | |  | |  | |  | |  | |  | |  | |  | |  | |  | |  | | **S** | |  | | **R** | | **K** | |  | |  | |  | |  | | **E** | |  | |  | |  |
| **Sw/GX/FS2/05(H9N2)** | **G1-like** | | |  | |  | |  | |  | |  | |  | |  | |  | |  | |  | |  | |  | | **S** | |  | | **R** | | **K** | |  | |  | |  | |  | | **E** | |  | |  | |  |
| **Ck/SH/F/98(H9N2)** | **SH/F/98-like** | | |  | |  | |  | |  | |  | |  | |  | |  | |  | |  | |  | |  | | **S** | |  | | **R** | | **E** | |  | |  | |  | |  | |  | |  | | **K** | |  |
| **Ck/HLJ/48/01(H9N2)** | **SH/F/98-like** | | |  | |  | |  | |  | |  | |  | |  | |  | |  | |  | |  | |  | | **S** | |  | | **R** | | **E** | |  | |  | |  | |  | |  | |  | | **K** | |  |
| **Ck/SH/10/01(H9N2)** | **SH/F/98-like** | | |  | |  | |  | |  | | **R** | |  | |  | |  | |  | |  | |  | |  | | **S** | |  | | **R** | | **E** | |  | |  | |  | |  | |  | |  | | **K** | |  |
| **Gs/GD/1/96(H5N1)** | **SH/F/98-like** | | |  | |  | |  | |  | |  | |  | |  | |  | | **S** | |  | |  | |  | | **S** | |  | | **R** | | **K** | |  | |  | |  | |  | |  | |  | |  | |  |
| **Dk/SH/35/02(H5N1)** | **SH/F/98-like** | | |  | |  | |  | |  | |  | |  | |  | |  | |  | |  | |  | |  | | **S** | |  | | **R** | | **E** | |  | |  | |  | |  | |  | |  | | **K** | |  |
| **Dk/HK/Y439/97(H9N2)** | **Y439-like** | | |  | |  | |  | |  | |  | |  | |  | |  | |  | |  | |  | |  | | **S** | |  | | **R** | | **K** | |  | |  | |  | |  | |  | |  | |  | |  |
| **Ck/KR/006/96(H9N2)** | **KR323-like** | | |  | |  | |  | |  | |  | |  | | **S** | |  | | **D** | |  | |  | |  | | **S** | |  | | **R** | | **K** | |  | |  | |  | |  | |  | |  | |  | |  |
| **Ck/KR/323/96(H9N2)** | **KR323-like** | | |  | |  | | **P** | |  | |  | |  | |  | |  | |  | |  | |  | |  | | **S** | |  | | **R** | | **K** | | **R** | |  | |  | |  | |  | |  | |  | |  |
| **Ck/KR/99029/99(H9N2)** | **KR323-like** | | |  | |  | |  | |  | |  | |  | |  | |  | |  | |  | |  | |  | | **S** | |  | | **R** | | **K** | |  | |  | |  | |  | |  | | **A** | | **K** | |  |
| **KR/0028/00(H9N2)** | **KR323-like** | | |  | |  | |  | |  | |  | |  | |  | |  | | **S** | | **M** | |  | |  | | **T** | |  | | **R** | | **K** | | **R** | |  | |  | |  | |  | |  | |  | |  |
| **Dk/DE/113/95(H9N2)** | **DE113-like** | | |  | |  | |  | |  | |  | |  | |  | |  | |  | |  | |  | |  | | **S** | |  | | **R** | | **K** | |  | |  | |  | |  | |  | |  | |  | |  |
| **Ph/IE/PV18/97(H9N2)** | **DE113-like** | | |  | |  | |  | |  | |  | |  | |  | |  | |  | |  | |  | |  | | **S** | |  | | **R** | | **K** | |  | |  | |  | |  | |  | |  | |  | |  |
| **DK/NC/8-174/00(H3N6)** | **DE113-like** | | |  | |  | |  | |  | |  | |  | |  | |  | |  | |  | |  | |  | | **S** | |  | | **R** | | **K** | |  | |  | |  | |  | |  | |  | |  | |  |
| **Ty/CA/189/66(H9N2)** | **WI/1/66-like** | | |  | |  | |  | | **K** | |  | |  | |  | |  | |  | |  | |  | | **D** | | **S** | |  | | **R** | | **K** | |  | |  | |  | |  | |  | |  | |  | |  |
| **Ty/WI/1/66(H9N2)** | **WI/1/66-like** | | |  | |  | |  | |  | |  | |  | |  | | **T** | | **S** | |  | |  | |  | | **P** | |  | | **R** | | **K** | |  | |  | |  | |  | |  | |  | |  | |  |

| **virus** | **lineage** | | **Amino acid residues at different positions in the encoding regions of the PB1 protein** | | | | | | | | | | | | | | | | | | | | | | | | | | | | | | | | | | | | | | | | | | | | | |  |
| --- | --- | --- | --- | --- | --- | --- | --- | --- | --- | --- | --- | --- | --- | --- | --- | --- | --- | --- | --- | --- | --- | --- | --- | --- | --- | --- | --- | --- | --- | --- | --- | --- | --- | --- | --- | --- | --- | --- | --- | --- | --- | --- | --- | --- | --- | --- | --- | --- | --- |
|  |  |  | **431** | | **433** | | **455** | | **460** | | **461** | | **467** | | **478** | | **490** | | **499** | | **511** | | **517** | | **532** | | **535** | | **536** | | **551** | | **558** | | **567** | | **573** | | **574** | | **581** | | **584** | | **587** | | **591** | |  |
| **BbM/GX/29/05(H9N2)** | | **H5N1-like** | | **Y** | | **K** | | **N** | | **Q** | | **A** | | **Y** | | **S** | | **F** | | **Y** | | **S** | | **I** | | **N** | | **I** | | **N** | | **F** | | **T** | | **Q** | | **S** | | **F** | | **E** | | **R** | | **A** | | **V** | |
| **BbM/GX/30/05(H9N2)** | | **H5N1-like** | |  | |  | |  | |  | |  | |  | |  | |  | |  | |  | |  | |  | |  | |  | |  | |  | |  | |  | |  | |  | |  | |  | |  | |
| **BbM/GX/31/05(H9N2)** | | **H5N1-like** | |  | |  | |  | |  | |  | |  | |  | |  | |  | |  | |  | |  | |  | |  | |  | |  | |  | |  | |  | |  | |  | |  | |  | |
| **Bird/GX/62/05(H9N2)** | | **H5N1-like** | |  | |  | |  | |  | |  | |  | |  | |  | |  | |  | |  | |  | |  | |  | |  | |  | | **K** | |  | |  | |  | |  | | **V** | |  | |
| **Qa/GX/B1/06(H9N2)** | | **H5N1-like** | |  | |  | |  | |  | |  | |  | |  | |  | |  | |  | |  | |  | |  | |  | |  | |  | |  | |  | |  | |  | |  | |  | |  | |
| **Bird/GX/H1/06(H9N2)** | | **H5N1-like** | |  | |  | |  | |  | |  | |  | |  | |  | |  | |  | |  | |  | |  | |  | |  | |  | |  | |  | |  | |  | |  | |  | |  | |
| **Sw/GX/S11/05(H9N2)** | | **H5N1-like** | |  | |  | |  | |  | |  | |  | |  | |  | |  | |  | |  | |  | |  | |  | |  | |  | |  | |  | |  | |  | |  | |  | |  | |
| **Sw/GX/S15/05(H9N2)** | | **H5N1-like** | |  | |  | |  | |  | |  | |  | |  | |  | |  | | **C** | |  | |  | |  | |  | |  | |  | |  | |  | |  | |  | |  | |  | |  | |
| **Ck/HK/YU22/02(H5N1)** | | **H5N1-like** | | **C** | |  | |  | |  | |  | |  | |  | |  | |  | |  | |  | |  | |  | |  | |  | |  | |  | |  | |  | |  | |  | |  | |  | |
| **Ck/YN/1252/03(H5N1)** | | **H5N1-like** | |  | |  | |  | |  | |  | |  | |  | |  | |  | |  | |  | |  | |  | |  | |  | |  | |  | |  | |  | |  | |  | |  | |  | |
| **Ck/HeN/01/04(H5N1)** | | **H5N1-like** | |  | |  | |  | |  | |  | |  | |  | |  | |  | |  | |  | |  | |  | |  | |  | |  | |  | |  | |  | |  | |  | |  | |  | |
| **Ck/BJ/1/94(H9N2)** | | **BJ94-like** | |  | |  | |  | |  | |  | |  | |  | |  | | **H** | |  | |  | |  | |  | |  | |  | |  | |  | |  | |  | |  | |  | |  | |  | |
| **Ck/HK/739/94(H9N2)** | | **BJ94-like** | |  | |  | |  | |  | |  | |  | |  | |  | |  | |  | |  | | **T** | |  | |  | |  | |  | |  | |  | |  | |  | |  | |  | |  | |
| **Ck/SD/6/96(H9N2)** | | **BJ94-like** | |  | |  | |  | |  | |  | |  | |  | |  | |  | |  | |  | |  | |  | |  | |  | |  | |  | |  | |  | |  | |  | |  | |  | |
| **Dk/NJ/2/97(H9N2)** | | **BJ94-like** | |  | |  | |  | |  | |  | |  | |  | |  | |  | |  | |  | |  | |  | |  | |  | |  | |  | |  | |  | |  | |  | |  | |  | |
| **Dk/HK/Y280/97(H9N2)** | | **BJ94-like** | |  | |  | |  | |  | |  | |  | |  | |  | |  | |  | |  | |  | |  | |  | |  | | **H** | |  | |  | |  | | **K** | |  | |  | |  | |
| **Ck/BJ/8/98(H9N2)** | | **BJ94-like** | |  | |  | |  | |  | |  | |  | |  | |  | |  | |  | |  | |  | |  | |  | |  | |  | |  | |  | |  | |  | |  | |  | |  | |
| **Ck/FJ/25/00(H9N2)** | | **BJ94-like** | |  | |  | |  | |  | |  | |  | |  | |  | |  | |  | |  | |  | |  | |  | |  | |  | |  | |  | |  | |  | |  | |  | |  | |
| **Ck/GD/4/00(H9N2)** | | **BJ94-like** | |  | |  | |  | |  | |  | |  | |  | |  | |  | |  | |  | |  | |  | |  | |  | |  | |  | |  | |  | |  | |  | |  | |  | |
| **Pg/NC/2-0461/00(H9N2)** | | **BJ94-like** | |  | |  | |  | |  | |  | |  | |  | |  | |  | |  | | **V** | |  | |  | |  | |  | |  | |  | |  | |  | |  | |  | |  | |  | |
| **Qa/NC/2-0460/00(H9N2)** | | **BJ94-like** | |  | |  | |  | |  | |  | |  | |  | |  | |  | |  | |  | |  | |  | |  | |  | |  | |  | |  | |  | |  | |  | |  | |  | |
| **WD/NC/2-0480/00(H9N2)** | | **BJ94-like** | |  | |  | |  | |  | |  | |  | |  | |  | |  | |  | | **V** | |  | |  | |  | |  | |  | |  | |  | |  | |  | |  | |  | |  | |
| **Ck/HeN/43/02(H9N2)** | | **BJ94-like** | |  | |  | |  | |  | |  | |  | |  | |  | |  | |  | |  | |  | |  | |  | |  | |  | |  | |  | |  | |  | |  | |  | |  | |
| **Ck/GD/6/97(H9N2)** | | **G1-like** | |  | |  | |  | |  | |  | |  | |  | |  | |  | |  | |  | |  | |  | |  | |  | |  | |  | |  | |  | |  | |  | |  | |  | |
| **Ck/HK/G23/97(H9N2)** | | **G1-like** | |  | |  | |  | |  | |  | |  | |  | |  | |  | |  | |  | |  | |  | |  | | **V** | |  | |  | |  | |  | |  | |  | |  | |  | |
| **Ck/HK/G9/97(H9N2)** | | **G1-like** | |  | |  | |  | |  | |  | |  | |  | |  | |  | |  | |  | |  | |  | |  | |  | |  | |  | |  | |  | |  | |  | |  | |  | |
| **Pg/HK/Y233/97(H9N2)** | | **G1-like** | |  | |  | |  | |  | |  | |  | |  | |  | |  | |  | |  | |  | |  | |  | | **V** | |  | |  | |  | |  | |  | |  | |  | |  | |
| **Qa/HK/G1/97(H9N2)** | | **G1-like** | |  | |  | |  | |  | |  | |  | |  | |  | |  | |  | |  | |  | |  | |  | |  | |  | |  | |  | |  | |  | |  | |  | |  | |
| **Pa/Chiba/1/97(H9N2)** | | **G1-like** | |  | |  | |  | |  | |  | |  | |  | |  | |  | |  | |  | |  | |  | |  | |  | |  | |  | |  | |  | |  | |  | |  | |  | |
| **Pa/Narita/92A/98(H9N2)** | | **G1-like** | |  | |  | |  | |  | |  | |  | |  | |  | |  | |  | |  | |  | |  | |  | |  | |  | |  | |  | |  | |  | |  | |  | |  | |
| **Ck/GX/10/99(H9N2)** | | **G1-like** | |  | |  | |  | |  | |  | |  | |  | |  | |  | |  | |  | |  | |  | |  | |  | |  | |  | |  | |  | |  | |  | |  | |  | |
| **Ck/GX/9/99(H9N2)** | | **G1-like** | |  | |  | |  | |  | |  | |  | |  | |  | |  | |  | |  | |  | |  | |  | |  | |  | |  | |  | |  | |  | |  | |  | |  | |
| **Ck/Pk/2/99(H9N2)** | | **G1-like** | |  | |  | |  | |  | |  | |  | |  | |  | |  | |  | |  | |  | |  | |  | |  | |  | |  | |  | |  | | **D** | |  | |  | |  | |
| **Ck/HLJ/35/00(H9N2)** | | **G1-like** | |  | |  | |  | |  | |  | |  | |  | |  | |  | |  | |  | |  | |  | |  | |  | |  | |  | |  | |  | |  | |  | |  | |  | |
| **HK/1073/99(H9N2)** | | **G1-like** | |  | |  | |  | |  | |  | |  | |  | |  | |  | |  | |  | |  | |  | |  | |  | |  | |  | |  | |  | |  | |  | |  | |  | |
| **HK/1074/99(H9N2)** | | **G1-like** | |  | |  | |  | |  | | **S** | |  | |  | |  | |  | |  | |  | |  | |  | |  | |  | |  | |  | |  | |  | |  | |  | |  | |  | |
| **GZ/333/99(H9N2)** | | **G1-like** | |  | |  | |  | |  | |  | |  | |  | |  | |  | |  | |  | |  | |  | |  | |  | |  | |  | |  | |  | |  | |  | |  | |  | |
| **HK/2108/03(H9N2)** | | **G1-like** | |  | |  | |  | |  | |  | |  | |  | |  | |  | |  | |  | |  | |  | |  | |  | |  | |  | |  | |  | |  | |  | |  | |  | |
| **Sw/GX/FS2/05(H9N2)** | | **G1-like** | |  | |  | |  | |  | |  | |  | |  | |  | |  | |  | |  | |  | |  | |  | |  | |  | | **K** | |  | |  | |  | |  | | **V** | |  | |
| **Ck/SH/F/98(H9N2)** | | **SH/F/98-like** | |  | | **R** | |  | |  | |  | |  | |  | |  | |  | |  | |  | |  | |  | |  | |  | |  | |  | | **A** | |  | |  | |  | |  | |  | |
| **Ck/HLJ/48/01(H9N2)** | | **SH/F/98-like** | |  | |  | |  | |  | |  | |  | |  | |  | |  | |  | |  | |  | |  | |  | |  | |  | |  | | **A** | |  | |  | |  | |  | |  | |
| **Ck/SH/10/01(H9N2)** | | **SH/F/98-like** | |  | |  | |  | |  | |  | |  | |  | |  | |  | |  | |  | |  | |  | |  | |  | |  | |  | | **A** | |  | |  | |  | |  | |  | |
| **Gs/GD/1/96(H5N1)** | | **SH/F/98-like** | |  | |  | |  | | **E** | |  | |  | | **T** | | **C** | |  | |  | |  | |  | | **M** | | **D** | |  | | **P** | |  | |  | |  | |  | |  | |  | |  | |
| **Dk/SH/35/02(H5N1)** | | **SH/F/98-like** | |  | |  | |  | |  | |  | |  | |  | |  | |  | |  | |  | |  | |  | |  | |  | |  | |  | | **A** | |  | |  | |  | |  | |  | |
| **Dk/HK/Y439/97(H9N2)** | | **Y439-like** | |  | |  | |  | |  | |  | |  | |  | |  | |  | |  | |  | |  | |  | |  | |  | |  | |  | |  | |  | |  | | **H** | | **T** | |  | |
| **Ck/KR/006/96(H9N2)** | | **KR323-like** | |  | |  | |  | |  | |  | |  | |  | |  | |  | |  | |  | |  | |  | |  | |  | |  | |  | |  | |  | |  | |  | |  | |  | |
| **Ck/KR/323/96(H9N2)** | | **KR323-like** | |  | |  | |  | |  | |  | |  | |  | |  | |  | |  | |  | |  | |  | |  | |  | |  | |  | |  | |  | |  | |  | |  | |  | |
| **Ck/KR/99029/99(H9N2)** | | **KR323-like** | |  | |  | |  | |  | |  | | **F** | |  | |  | |  | |  | |  | | **T** | |  | |  | |  | |  | |  | |  | | **L** | |  | |  | |  | |  | |
| **KR/0028/00(H9N2)** | | **KR323-like** | |  | |  | |  | |  | |  | |  | |  | |  | |  | |  | |  | |  | |  | |  | |  | |  | |  | |  | |  | | **V** | |  | |  | |  | |
| **Dk/DE/113/95(H9N2)** | | **DE113-like** | |  | |  | |  | |  | |  | |  | |  | |  | |  | |  | |  | |  | |  | |  | |  | |  | |  | |  | |  | |  | |  | |  | |  | |
| **Ph/IE/PV18/97(H9N2)** | | **DE113-like** | |  | |  | |  | |  | |  | |  | |  | |  | |  | |  | |  | |  | |  | |  | |  | |  | |  | |  | |  | |  | |  | | **T** | | **I** | |
| **DK/NC/8-174/00(H3N6)** | | **DE113-like** | |  | |  | |  | |  | |  | |  | |  | |  | |  | |  | |  | |  | |  | |  | |  | |  | |  | |  | |  | |  | |  | |  | |  | |
| **Ty/CA/189/66(H9N2)** | | **WI/1/66-like** | |  | |  | |  | |  | |  | |  | |  | |  | |  | |  | |  | |  | |  | |  | |  | |  | |  | |  | |  | |  | |  | |  | |  | |
| **Ty/WI/1/66(H9N2)** | | **WI/1/66-like** | |  | |  | | **D** | |  | |  | |  | |  | |  | |  | |  | |  | |  | |  | |  | |  | |  | |  | |  | |  | |  | |  | |  | |  | |

| **virus** | **lineage** | | **Amino acid residues at different positions in the encoding regions of the PB1 protein** | | | | | | | | | | | | | | | | | | | | | | | | | | | | | | | | | | | | | | | | | | | | | |  |
| --- | --- | --- | --- | --- | --- | --- | --- | --- | --- | --- | --- | --- | --- | --- | --- | --- | --- | --- | --- | --- | --- | --- | --- | --- | --- | --- | --- | --- | --- | --- | --- | --- | --- | --- | --- | --- | --- | --- | --- | --- | --- | --- | --- | --- | --- | --- | --- | --- | --- |
|  |  |  | **594** | | **598** | | **609** | | **610** | | **611** | | **612** | | **613** | | **614** | | **618** | | **621** | | **635** | | **637** | | **638** | | **642** | | **643** | | **653** | | **654** | | **661** | | **667** | | **670** | | **694** | | **696** | | **698** | |  |
| **BbM/GX/29/05(H9N2)** | | **H5N1-like** | | **G** | | **L** | | **V** | | **C** | | **L** | | **K** | | **W** | | **E** | | **E** | | **Q** | | **K** | | **I** | | **E** | | **N** | | **A** | | **K** | | **S** | | **A** | | **I** | | **R** | | **N** | | **F** | | **K** | |
| **BbM/GX/30/05(H9N2)** | | **H5N1-like** | |  | |  | |  | |  | |  | |  | |  | |  | |  | |  | |  | |  | |  | |  | |  | |  | |  | |  | |  | |  | |  | |  | |  | |
| **BbM/GX/31/05(H9N2)** | | **H5N1-like** | |  | |  | |  | |  | |  | |  | |  | |  | |  | |  | |  | |  | |  | |  | |  | |  | |  | |  | |  | |  | |  | |  | |  | |
| **Bird/GX/62/05(H9N2)** | | **H5N1-like** | |  | |  | |  | |  | |  | |  | |  | |  | |  | |  | |  | | **V** | |  | |  | |  | |  | |  | |  | |  | |  | |  | |  | |  | |
| **Qa/GX/B1/06(H9N2)** | | **H5N1-like** | |  | |  | |  | |  | |  | |  | |  | |  | |  | |  | |  | |  | |  | |  | |  | |  | |  | |  | |  | |  | |  | |  | |  | |
| **Bird/GX/H1/06(H9N2)** | | **H5N1-like** | |  | |  | |  | |  | |  | |  | |  | |  | |  | |  | |  | |  | |  | |  | |  | |  | |  | |  | |  | |  | |  | |  | |  | |
| **Sw/GX/S11/05(H9N2)** | | **H5N1-like** | |  | |  | |  | |  | |  | |  | |  | |  | |  | |  | |  | |  | |  | |  | |  | |  | |  | |  | |  | |  | |  | |  | |  | |
| **Sw/GX/S15/05(H9N2)** | | **H5N1-like** | |  | |  | |  | |  | |  | |  | |  | |  | |  | |  | |  | |  | |  | |  | |  | |  | |  | |  | |  | |  | |  | |  | |  | |
| **Ck/HK/YU22/02(H5N1)** | | **H5N1-like** | |  | |  | |  | |  | |  | |  | |  | |  | |  | |  | |  | |  | |  | |  | |  | |  | |  | |  | |  | |  | |  | |  | |  | |
| **Ck/YN/1252/03(H5N1)** | | **H5N1-like** | |  | |  | |  | |  | |  | |  | |  | |  | |  | |  | |  | |  | |  | |  | |  | |  | |  | |  | |  | |  | |  | |  | |  | |
| **Ck/HeN/01/04(H5N1)** | | **H5N1-like** | |  | |  | |  | |  | |  | |  | |  | |  | |  | |  | |  | |  | |  | |  | |  | |  | |  | |  | |  | |  | |  | |  | |  | |
| **Ck/BJ/1/94(H9N2)** | | **BJ94-like** | |  | |  | |  | |  | |  | | **N** | | **G** | | **S** | |  | |  | |  | |  | |  | |  | |  | |  | |  | |  | |  | |  | |  | |  | |  | |
| **Ck/HK/739/94(H9N2)** | | **BJ94-like** | |  | |  | |  | |  | |  | |  | |  | |  | |  | | **K** | |  | |  | |  | |  | |  | |  | |  | |  | |  | |  | |  | |  | |  | |
| **Ck/SD/6/96(H9N2)** | | **BJ94-like** | |  | |  | |  | |  | |  | |  | |  | |  | |  | | **K** | |  | |  | |  | |  | |  | |  | |  | |  | |  | |  | |  | |  | |  | |
| **Dk/NJ/2/97(H9N2)** | | **BJ94-like** | |  | |  | |  | |  | |  | |  | |  | |  | |  | | **K** | |  | |  | |  | |  | |  | |  | |  | |  | |  | |  | |  | |  | |  | |
| **Dk/HK/Y280/97(H9N2)** | | **BJ94-like** | | **E** | |  | |  | |  | |  | |  | |  | |  | |  | | **K** | |  | |  | |  | |  | |  | |  | |  | |  | |  | |  | |  | |  | |  | |
| **Ck/BJ/8/98(H9N2)** | | **BJ94-like** | |  | |  | |  | |  | |  | |  | |  | |  | |  | | **K** | |  | |  | |  | |  | |  | |  | |  | |  | |  | |  | |  | |  | |  | |
| **Ck/FJ/25/00(H9N2)** | | **BJ94-like** | |  | |  | |  | |  | |  | |  | |  | |  | |  | | **K** | |  | |  | |  | |  | |  | |  | |  | |  | | **V** | |  | |  | |  | |  | |
| **Ck/GD/4/00(H9N2)** | | **BJ94-like** | |  | |  | |  | |  | |  | |  | |  | |  | |  | | **K** | |  | |  | |  | |  | |  | |  | |  | |  | |  | |  | | **I** | |  | |  | |
| **Pg/NC/2-0461/00(H9N2)** | | **BJ94-like** | |  | |  | |  | |  | |  | |  | |  | |  | |  | | **K** | |  | |  | |  | |  | |  | |  | |  | |  | |  | |  | |  | |  | |  | |
| **Qa/NC/2-0460/00(H9N2)** | | **BJ94-like** | |  | |  | |  | |  | |  | |  | |  | |  | |  | | **K** | |  | |  | |  | |  | | **T** | |  | |  | |  | |  | |  | |  | |  | |  | |
| **WD/NC/2-0480/00(H9N2)** | | **BJ94-like** | |  | |  | |  | |  | |  | |  | |  | |  | |  | | **K** | |  | |  | |  | |  | |  | |  | |  | |  | |  | |  | |  | |  | |  | |
| **Ck/HeN/43/02(H9N2)** | | **BJ94-like** | |  | |  | |  | |  | |  | |  | |  | |  | |  | | **K** | |  | |  | |  | |  | |  | |  | |  | |  | |  | |  | |  | |  | |  | |
| **Ck/GD/6/97(H9N2)** | | **G1-like** | |  | |  | |  | |  | |  | |  | |  | |  | |  | |  | |  | | **V** | |  | |  | |  | |  | |  | |  | |  | |  | | **T** | |  | |  | |
| **Ck/HK/G23/97(H9N2)** | | **G1-like** | |  | |  | |  | |  | |  | |  | |  | | **D** | |  | |  | |  | | **V** | |  | |  | |  | |  | |  | |  | |  | |  | | **T** | |  | |  | |
| **Ck/HK/G9/97(H9N2)** | | **G1-like** | |  | |  | |  | | **A** | |  | |  | |  | | **D** | |  | |  | |  | | **V** | |  | |  | |  | |  | |  | |  | |  | |  | | **T** | |  | |  | |
| **Pg/HK/Y233/97(H9N2)** | | **G1-like** | |  | |  | |  | |  | |  | |  | |  | |  | |  | |  | |  | | **V** | |  | |  | |  | |  | |  | |  | |  | |  | | **T** | |  | |  | |
| **Qa/HK/G1/97(H9N2)** | | **G1-like** | |  | |  | |  | | **G** | |  | |  | |  | |  | |  | |  | |  | | **V** | |  | |  | |  | |  | |  | |  | |  | |  | | **T** | |  | |  | |
| **Pa/Chiba/1/97(H9N2)** | | **G1-like** | |  | |  | |  | |  | |  | |  | |  | |  | |  | |  | |  | | **V** | |  | |  | |  | |  | |  | |  | |  | |  | | **T** | |  | |  | |
| **Pa/Narita/92A/98(H9N2)** | | **G1-like** | |  | |  | |  | |  | |  | |  | |  | |  | |  | |  | |  | | **V** | |  | |  | |  | |  | | **N** | |  | |  | |  | | **T** | |  | |  | |
| **Ck/GX/10/99(H9N2)** | | **G1-like** | |  | |  | |  | |  | | **S** | |  | |  | |  | |  | |  | |  | | **V** | |  | |  | |  | |  | |  | |  | |  | |  | | **T** | |  | |  | |
| **Ck/GX/9/99(H9N2)** | | **G1-like** | |  | |  | |  | |  | | **S** | |  | |  | |  | |  | |  | |  | | **V** | |  | |  | |  | |  | |  | |  | |  | |  | | **T** | |  | |  | |
| **Ck/Pk/2/99(H9N2)** | | **G1-like** | |  | |  | |  | |  | |  | |  | |  | |  | |  | |  | |  | | **V** | |  | |  | |  | |  | |  | |  | |  | |  | | **T** | |  | |  | |
| **Ck/HLJ/35/00(H9N2)** | | **G1-like** | |  | |  | |  | |  | |  | |  | |  | |  | |  | |  | |  | | **V** | |  | |  | |  | |  | |  | |  | |  | |  | | **T** | |  | |  | |
| **HK/1073/99(H9N2)** | | **G1-like** | |  | |  | |  | |  | |  | |  | |  | |  | |  | |  | |  | | **V** | |  | |  | |  | |  | |  | |  | |  | |  | | **T** | |  | |  | |
| **HK/1074/99(H9N2)** | | **G1-like** | |  | |  | |  | |  | |  | |  | |  | |  | |  | |  | |  | | **V** | |  | |  | |  | |  | |  | |  | |  | |  | | **T** | |  | |  | |
| **GZ/333/99(H9N2)** | | **G1-like** | |  | |  | |  | |  | |  | |  | |  | |  | |  | |  | |  | | **V** | |  | |  | |  | |  | |  | |  | |  | |  | | **T** | |  | |  | |
| **HK/2108/03(H9N2)** | | **G1-like** | |  | |  | |  | |  | |  | |  | |  | |  | |  | |  | |  | | **V** | |  | |  | |  | |  | |  | |  | |  | |  | | **T** | |  | |  | |
| **Sw/GX/FS2/05(H9N2)** | | **G1-like** | |  | |  | |  | |  | |  | |  | |  | |  | |  | |  | |  | | **V** | |  | |  | |  | |  | |  | |  | |  | | **G** | | **T** | | **S** | |  | |
| **Ck/SH/F/98(H9N2)** | | **SH/F/98-like** | |  | |  | |  | |  | |  | |  | |  | |  | |  | |  | |  | |  | | **D** | |  | |  | |  | |  | |  | |  | |  | |  | |  | |  | |
| **Ck/HLJ/48/01(H9N2)** | | **SH/F/98-like** | |  | |  | |  | |  | |  | |  | |  | |  | | **D** | |  | |  | |  | | **D** | | **Y** | |  | |  | |  | |  | |  | |  | |  | |  | | **E** | |
| **Ck/SH/10/01(H9N2)** | | **SH/F/98-like** | |  | |  | |  | |  | |  | |  | |  | |  | |  | |  | | **E** | |  | | **D** | |  | |  | |  | |  | |  | |  | |  | |  | |  | |  | |
| **Gs/GD/1/96(H5N1)** | | **SH/F/98-like** | |  | | **P** | | **A** | | **G** | |  | |  | |  | |  | |  | |  | |  | |  | |  | |  | |  | |  | |  | |  | |  | |  | |  | |  | |  | |
| **Dk/SH/35/02(H5N1)** | | **SH/F/98-like** | |  | |  | |  | |  | |  | |  | |  | |  | |  | |  | |  | |  | | **D** | |  | |  | |  | |  | |  | |  | |  | |  | |  | |  | |
| **Dk/HK/Y439/97(H9N2)** | | **Y439-like** | |  | |  | |  | |  | |  | |  | |  | |  | |  | |  | |  | |  | |  | |  | |  | |  | |  | |  | |  | |  | |  | |  | |  | |
| **Ck/KR/006/96(H9N2)** | | **KR323-like** | |  | |  | |  | |  | |  | |  | |  | |  | |  | |  | |  | |  | |  | |  | |  | |  | |  | |  | |  | |  | |  | |  | |  | |
| **Ck/KR/323/96(H9N2)** | | **KR323-like** | |  | |  | |  | |  | |  | |  | |  | |  | |  | |  | |  | |  | |  | |  | |  | |  | |  | |  | |  | |  | |  | |  | |  | |
| **Ck/KR/99029/99(H9N2)** | | **KR323-like** | |  | |  | |  | |  | |  | |  | |  | |  | |  | |  | |  | |  | |  | |  | |  | |  | |  | |  | |  | |  | |  | |  | |  | |
| **KR/0028/00(H9N2)** | | **KR323-like** | |  | |  | |  | |  | |  | |  | |  | |  | |  | |  | |  | |  | |  | |  | |  | |  | |  | |  | |  | |  | |  | |  | |  | |
| **Dk/DE/113/95(H9N2)** | | **DE113-like** | |  | |  | |  | |  | |  | |  | |  | |  | |  | |  | |  | |  | |  | |  | |  | |  | |  | |  | |  | |  | |  | |  | |  | |
| **Ph/IE/PV18/97(H9N2)** | | **DE113-like** | |  | |  | |  | |  | |  | |  | |  | |  | |  | |  | |  | |  | |  | |  | |  | |  | |  | |  | |  | |  | |  | |  | |  | |
| **DK/NC/8-174/00(H3N6)** | | **DE113-like** | |  | |  | |  | |  | |  | |  | |  | |  | |  | |  | |  | |  | |  | |  | |  | |  | |  | |  | |  | |  | |  | |  | |  | |
| **Ty/CA/189/66(H9N2)** | | **WI/1/66-like** | |  | |  | |  | |  | |  | |  | |  | |  | |  | |  | |  | |  | |  | |  | |  | | **R** | |  | | **T** | |  | |  | |  | |  | |  | |
| **Ty/WI/1/66(H9N2)** | | **WI/1/66-like** | |  | |  | |  | |  | |  | |  | |  | |  | |  | |  | |  | |  | |  | |  | |  | |  | |  | |  | |  | |  | |  | |  | |  | |

| **virus** | **lineage** | | **Amino acid residues at different positions in the encoding regions of the PB1 protein** | | | | | | | | | | | | | | | | | | | | | | | | | | | | | | | | | | | | | | | | | | | | | |  |
| --- | --- | --- | --- | --- | --- | --- | --- | --- | --- | --- | --- | --- | --- | --- | --- | --- | --- | --- | --- | --- | --- | --- | --- | --- | --- | --- | --- | --- | --- | --- | --- | --- | --- | --- | --- | --- | --- | --- | --- | --- | --- | --- | --- | --- | --- | --- | --- | --- | --- |
|  |  |  | **702** | | **715** | | **719** | | **722** | | **729** | | **735** | | **739** | | **740** | | **742** | | **744** | | **747** | | **753** | | **754** | | **756** | | **757** | |  | |  | |  | |  | |  | |  | |  | |  | |  |
| **BbM/GX/29/05(H9N2)** | | **H5N1-like** | | **S** | | **V** | | **V** | | **A** | | **D** | | **I** | | **E** | | **F** | | **E** | | **M** | | **C** | | **L** | | **R** | | **Q** | | **N** | |  | |  | |  | |  | |  | |  | |  | |  | |
| **BbM/GX/30/05(H9N2)** | | **H5N1-like** | |  | |  | |  | |  | |  | |  | |  | |  | |  | |  | |  | |  | |  | |  | |  | |  | |  | |  | |  | |  | |  | |  | |  | |
| **BbM/GX/31/05(H9N2)** | | **H5N1-like** | |  | |  | |  | |  | |  | |  | |  | |  | |  | |  | |  | |  | |  | |  | |  | |  | |  | |  | |  | |  | |  | |  | |  | |
| **Bird/GX/62/05(H9N2)** | | **H5N1-like** | |  | |  | |  | |  | |  | |  | |  | |  | |  | |  | |  | |  | |  | |  | |  | |  | |  | |  | |  | |  | |  | |  | |  | |
| **Qa/GX/B1/06(H9N2)** | | **H5N1-like** | |  | |  | |  | |  | |  | |  | |  | |  | |  | |  | |  | |  | |  | |  | |  | |  | |  | |  | |  | |  | |  | |  | |  | |
| **Bird/GX/H1/06(H9N2)** | | **H5N1-like** | |  | |  | |  | |  | |  | |  | |  | |  | |  | |  | |  | |  | |  | |  | |  | |  | |  | |  | |  | |  | |  | |  | |  | |
| **Sw/GX/S11/05(H9N2)** | | **H5N1-like** | |  | |  | |  | |  | |  | |  | |  | |  | | **G** | |  | |  | |  | |  | |  | |  | |  | |  | |  | |  | |  | |  | |  | |  | |
| **Sw/GX/S15/05(H9N2)** | | **H5N1-like** | |  | |  | |  | |  | |  | |  | |  | |  | |  | |  | |  | |  | |  | |  | |  | |  | |  | |  | |  | |  | |  | |  | |  | |
| **Ck/HK/YU22/02(H5N1)** | | **H5N1-like** | |  | |  | |  | |  | |  | |  | |  | |  | |  | |  | |  | |  | |  | |  | | **K** | |  | |  | |  | |  | |  | |  | |  | |  | |
| **Ck/YN/1252/03(H5N1)** | | **H5N1-like** | |  | |  | |  | |  | |  | |  | |  | |  | |  | |  | |  | |  | |  | |  | | **K** | |  | |  | |  | |  | |  | |  | |  | |  | |
| **Ck/HeN/01/04(H5N1)** | | **H5N1-like** | |  | |  | |  | |  | |  | |  | |  | |  | |  | |  | |  | |  | |  | |  | | **K** | |  | |  | |  | |  | |  | |  | |  | |  | |
| **Ck/BJ/1/94(H9N2)** | | **BJ94-like** | |  | |  | |  | |  | |  | |  | |  | |  | |  | |  | |  | |  | |  | |  | |  | |  | |  | |  | |  | |  | |  | |  | |  | |
| **Ck/HK/739/94(H9N2)** | | **BJ94-like** | |  | |  | |  | |  | | **G** | |  | |  | | **V** | |  | | **T** | | ***** | | ***** | | ***** | | ***** | | ***** | |  | |  | |  | |  | |  | |  | |  | |  | |
| **Ck/SD/6/96(H9N2)** | | **BJ94-like** | |  | |  | |  | |  | |  | |  | |  | |  | |  | | **T** | |  | |  | |  | |  | | **K** | |  | |  | |  | |  | |  | |  | |  | |  | |
| **Dk/NJ/2/97(H9N2)** | | **BJ94-like** | |  | |  | |  | |  | |  | | **V** | |  | |  | |  | | **T** | |  | |  | |  | |  | | **K** | |  | |  | |  | |  | |  | |  | |  | |  | |
| **Dk/HK/Y280/97(H9N2)** | | **BJ94-like** | |  | |  | |  | |  | |  | |  | |  | | ***** | | ***** | | ***** | | ***** | | ***** | | ***** | | ***** | | ***** | |  | |  | |  | |  | |  | |  | |  | |  | |
| **Ck/BJ/8/98(H9N2)** | | **BJ94-like** | |  | |  | |  | |  | |  | |  | |  | |  | |  | | **T** | |  | |  | |  | | **H** | | **K** | |  | |  | |  | |  | |  | |  | |  | |  | |
| **Ck/FJ/25/00(H9N2)** | | **BJ94-like** | |  | |  | | **I** | |  | |  | |  | |  | |  | |  | | **T** | |  | |  | |  | |  | | **K** | |  | |  | |  | |  | |  | |  | |  | |  | |
| **Ck/GD/4/00(H9N2)** | | **BJ94-like** | |  | |  | |  | |  | |  | |  | |  | |  | |  | |  | |  | |  | |  | |  | | **K** | |  | |  | |  | |  | |  | |  | |  | |  | |
| **Pg/NC/2-0461/00(H9N2)** | | **BJ94-like** | |  | |  | |  | |  | |  | |  | |  | |  | |  | |  | |  | |  | |  | |  | | **K** | |  | |  | |  | |  | |  | |  | |  | |  | |
| **Qa/NC/2-0460/00(H9N2)** | | **BJ94-like** | |  | |  | |  | |  | |  | |  | |  | |  | |  | |  | |  | |  | |  | |  | | **K** | |  | |  | |  | |  | |  | |  | |  | |  | |
| **WD/NC/2-0480/00(H9N2)** | | **BJ94-like** | |  | |  | |  | |  | |  | |  | |  | |  | |  | |  | |  | |  | |  | |  | | **K** | |  | |  | |  | |  | |  | |  | |  | |  | |
| **Ck/HeN/43/02(H9N2)** | | **BJ94-like** | |  | |  | |  | |  | |  | |  | |  | |  | |  | |  | |  | |  | |  | |  | | **K** | |  | |  | |  | |  | |  | |  | |  | |  | |
| **Ck/GD/6/97(H9N2)** | | **G1-like** | |  | | **M** | |  | |  | |  | |  | |  | |  | |  | | **L** | |  | |  | | **G** | |  | | **G** | |  | |  | |  | |  | |  | |  | |  | |  | |
| **Ck/HK/G23/97(H9N2)** | | **G1-like** | |  | | **M** | |  | |  | |  | |  | | **A** | | **Y** | | **N** | | ***** | | ***** | | ***** | | ***** | | ***** | | ***** | |  | |  | |  | |  | |  | |  | |  | |  | |
| **Ck/HK/G9/97(H9N2)** | | **G1-like** | |  | | **M** | |  | |  | |  | |  | |  | |  | |  | | **L** | | **W** | | ***** | | ***** | | ***** | | ***** | |  | |  | |  | |  | |  | |  | |  | |  | |
| **Pg/HK/Y233/97(H9N2)** | | **G1-like** | |  | | **M** | |  | |  | |  | |  | | **A** | | **Y** | | **N** | | ***** | | ***** | | ***** | | ***** | | ***** | | ***** | |  | |  | |  | |  | |  | |  | |  | |  | |
| **Qa/HK/G1/97(H9N2)** | | **G1-like** | |  | | **M** | |  | |  | |  | |  | |  | |  | |  | | **L** | | ***** | | ***** | | ***** | | ***** | | ***** | |  | |  | |  | |  | |  | |  | |  | |  | |
| **Pa/Chiba/1/97(H9N2)** | | **G1-like** | |  | | **M** | |  | |  | |  | |  | |  | |  | |  | | **L** | |  | |  | | **G** | |  | | **G** | |  | |  | |  | |  | |  | |  | |  | |  | |
| **Pa/Narita/92A/98(H9N2)** | | **G1-like** | |  | | **M** | |  | |  | |  | |  | |  | |  | |  | | **L** | |  | |  | | **G** | |  | | **G** | |  | |  | |  | |  | |  | |  | |  | |  | |
| **Ck/GX/10/99(H9N2)** | | **G1-like** | |  | | **M** | |  | |  | |  | |  | |  | |  | |  | | **L** | |  | |  | | **G** | |  | | **G** | |  | |  | |  | |  | |  | |  | |  | |  | |
| **Ck/GX/9/99(H9N2)** | | **G1-like** | |  | | **M** | |  | |  | |  | |  | |  | |  | |  | | **L** | |  | |  | | **G** | |  | | **G** | |  | |  | |  | |  | |  | |  | |  | |  | |
| **Ck/Pk/2/99(H9N2)** | | **G1-like** | |  | | **M** | |  | |  | |  | |  | |  | |  | |  | | **L** | |  | |  | | **G** | |  | | **G** | |  | |  | |  | |  | |  | |  | |  | |  | |
| **Ck/HLJ/35/00(H9N2)** | | **G1-like** | |  | | **M** | |  | |  | |  | |  | |  | |  | |  | | **L** | |  | |  | | **G** | |  | | **G** | |  | |  | |  | |  | |  | |  | |  | |  | |
| **HK/1073/99(H9N2)** | | **G1-like** | |  | | **M** | |  | |  | |  | |  | |  | |  | |  | | **L** | |  | |  | | **G** | |  | | **G** | |  | |  | |  | |  | |  | |  | |  | |  | |
| **HK/1074/99(H9N2)** | | **G1-like** | |  | | **M** | |  | |  | |  | |  | |  | |  | |  | | **L** | |  | |  | | **G** | |  | | **G** | |  | |  | |  | |  | |  | |  | |  | |  | |
| **GZ/333/99(H9N2)** | | **G1-like** | |  | | **M** | |  | |  | |  | |  | |  | |  | |  | | **L** | |  | |  | | **G** | |  | | **G** | |  | |  | |  | |  | |  | |  | |  | |  | |
| **HK/2108/03(H9N2)** | | **G1-like** | |  | | **M** | |  | |  | |  | |  | |  | |  | |  | | **L** | |  | |  | | **G** | |  | | **G** | |  | |  | |  | |  | |  | |  | |  | |  | |
| **Sw/GX/FS2/05(H9N2)** | | **G1-like** | | **N** | | **M** | |  | |  | |  | |  | |  | |  | |  | | **L** | |  | | **P** | | **G** | |  | | **G** | |  | |  | |  | |  | |  | |  | |  | |  | |
| **Ck/SH/F/98(H9N2)** | | **SH/F/98-like** | |  | |  | |  | |  | |  | |  | |  | |  | |  | |  | |  | |  | |  | |  | | **K** | |  | |  | |  | |  | |  | |  | |  | |  | |
| **Ck/HLJ/48/01(H9N2)** | | **SH/F/98-like** | |  | |  | |  | |  | |  | |  | |  | |  | |  | |  | |  | |  | |  | |  | | **K** | |  | |  | |  | |  | |  | |  | |  | |  | |
| **Ck/SH/10/01(H9N2)** | | **SH/F/98-like** | |  | |  | |  | |  | |  | |  | |  | |  | |  | |  | |  | |  | |  | |  | | **K** | |  | |  | |  | |  | |  | |  | |  | |  | |
| **Gs/GD/1/96(H5N1)** | | **SH/F/98-like** | |  | |  | |  | |  | |  | |  | |  | |  | |  | |  | |  | |  | | **G** | |  | | **K** | |  | |  | |  | |  | |  | |  | |  | |  | |
| **Dk/SH/35/02(H5N1)** | | **SH/F/98-like** | |  | |  | |  | |  | |  | |  | |  | |  | |  | |  | |  | |  | |  | |  | | **K** | |  | |  | |  | |  | |  | |  | |  | |  | |
| **Dk/HK/Y439/97(H9N2)** | | **Y439-like** | |  | |  | |  | |  | |  | |  | | **A** | | ***** | | ***** | | ***** | | ***** | | ***** | | ***** | | ***** | | ***** | |  | |  | |  | |  | |  | |  | |  | |  | |
| **Ck/KR/006/96(H9N2)** | | **KR323-like** | |  | |  | |  | |  | |  | |  | |  | |  | |  | |  | | ***** | | ***** | | ***** | | ***** | | ***** | |  | |  | |  | |  | |  | |  | |  | |  | |
| **Ck/KR/323/96(H9N2)** | | **KR323-like** | |  | |  | |  | |  | |  | |  | |  | |  | |  | |  | | ***** | | ***** | | ***** | | ***** | | ***** | |  | |  | |  | |  | |  | |  | |  | |  | |
| **Ck/KR/99029/99(H9N2)** | | **KR323-like** | |  | |  | |  | |  | |  | |  | |  | |  | |  | |  | |  | |  | |  | |  | | **K** | |  | |  | |  | |  | |  | |  | |  | |  | |
| **KR/0028/00(H9N2)** | | **KR323-like** | |  | |  | |  | |  | |  | |  | |  | | **L** | |  | |  | |  | |  | |  | |  | | **K** | |  | |  | |  | |  | |  | |  | |  | |  | |
| **Dk/DE/113/95(H9N2)** | | **DE113-like** | |  | |  | |  | |  | |  | |  | |  | |  | |  | |  | |  | |  | |  | |  | | ***** | |  | |  | |  | |  | |  | |  | |  | |  | |
| **Ph/IE/PV18/97(H9N2)** | | **DE113-like** | |  | |  | |  | |  | |  | |  | |  | |  | |  | |  | |  | | ***** | | ***** | | ***** | | ***** | |  | |  | |  | |  | |  | |  | |  | |  | |
| **DK/NC/8-174/00(H3N6)** | | **DE113-like** | |  | |  | |  | | **V** | |  | |  | |  | |  | |  | |  | |  | |  | |  | |  | | **K** | |  | |  | |  | |  | |  | |  | |  | |  | |
| **Ty/CA/189/66(H9N2)** | | **WI/1/66-like** | |  | |  | |  | |  | |  | |  | |  | |  | |  | |  | |  | | ***** | | ***** | | ***** | | ***** | |  | |  | |  | |  | |  | |  | |  | |  | |
| **Ty/WI/1/66(H9N2)** | | **WI/1/66-like** | |  | |  | |  | |  | |  | |  | |  | |  | |  | |  | |  | |  | |  | |  | | **K** | |  | |  | |  | |  | |  | |  | |  | |  | |

* **reprents not determined amino acid. Virus names and abbreviations are in the legend to Figure 1.**
